# Arts in Education: A Systematic Review of Competency Outcomes in Quasi-Experimental and Experimental Studies

**DOI:** 10.3389/fpsyg.2021.623935

**Published:** 2021-04-15

**Authors:** Verena Schneider, Anette Rohmann

**Affiliations:** Department of Community Psychology, FernUniversität in Hagen (University of Hagen), Hagen, Germany

**Keywords:** arts education, music, dance, drama, visual arts, systematic (literature) review, competencies

## Abstract

Arts education in schools frequently experiences the pressure of being validated by demonstrating quantitative impact on academic outcomes. The quantitative evidence to date has been characterized by the application of largely correlational designs and frequently applies a narrow focus on instrumental outcomes such as academically relevant competencies. The present review aims to summarize quantitative evidence from quasi-experimental and experimental studies with pre-test post-test designs on the effects of school-based arts education on a broader range of competency outcomes, including intra- and interindividual competencies. A systematic literature search was conducted to identify relevant evaluation studies. Twenty-four articles reporting on 26 evaluation studies were eligible for inclusion, and their results were reviewed in terms of art domains and outcome categories. Whilst there is some evidence of beneficial effects on some competencies, for example of music education on arithmetic abilities, speech segmentation and processing speed, the evidence across arts domains and for different outcomes is limited due to small sample sizes, small number of studies, and a large range of effect sizes. The review highlights that sufficiently powered (quasi-)experimental studies with pre-test post-test designs evaluating arts education are sparse and that the “gold standard” of experimental research comes at the expense of a number of other study characteristics such as sample size, intervention and follow-up length. By summarizing the limitations of the current (quasi-)experimental research, the application of experimental designs is critically assessed and a combination with qualitative methods in mixed-method designs and choice of relevant outcomes discussed.

## Introduction

Can the arts promote the creative skills needed for innovation? What are the beneficial effects of music training on computation skills, spatial performance, memory and reading skills? Do actors have better verbal and emotional skills? Can dance promote a sense of belonging? Does arts education improve educational attainment, attention and motivation?

Questions about effects of arts education on outcomes such as academic achievement and competencies have inspired much research in an effort to defend the arts' position in curricula. Qualitative, mixed-method and quantitative research support the notion that engagement in the arts can contribute to positive outcomes such as academic achievement, attainment, social behavior and social transformation alongside health benefits such as wellbeing (Deasy, 2002; Ewing, 2010; McLellan et al., 2012a, 2015; Winner et al., 2013; Fancourt and Finn, 2019). However, the so-called “gold standard” experimental research designs are rare, and findings on the causal impact on academically relevant outcomes have frequently been limited or inconclusive (e.g., Winner et al., 2013). Yet, experimental designs are often not suitable for educational evaluation studies and their ability to capture the complex experiences in arts learning have been questioned (Ewing, 2010). Further, small sample sizes and short follow-up periods are common in experimental studies and preclude confidence in conclusions. Finally, while the choice of instrumental outcomes such as children's performances in other subjects may seem attractive, the implications of an exclusive focus on such outcomes have been critically discussed (Winner et al., 2013). The current review therefore seeks, first, to summarize the evidence from quasi-experimental and experimental studies on a range of transferrable skills and competency outcomes in school-based arts education. Second, it seeks to describe and evaluate the methodological characteristics of the included studies and implications on conclusions.

According to Oxford University Press (2019), art is defined as the “expression or application of human creative skill and imagination, typically in a visual form such as painting or sculpture, producing works to be appreciated primarily for their beauty or emotional power” (1st paragraph), and the arts as the “various branches of creative activity, such as painting, music, literature, and dance” (2nd paragraph). These definitions are reflective of the arts as activities which involve creative problem solving and opportunities for expression. Arts engagement can further involve social interaction and collaboration and allow for an exploration of the self in an environment removed from the binary evaluation of performance as either right or wrong. The arts may therefore be well positioned to stimulate the development of intra- and interpersonal outcomes. For example, as McLellan et al. (2012b) argue using the theoretical framework of the self-determination theory (Deci and Ryan, 1985), the arts may offer opportunities for experiences of competence, autonomy and relatedness which in turn promote motivation and wellbeing.

### State of Research

Research into the impacts of arts education can be categorized by the type of arts experience or engagement. First, research can be categorized whether it evaluates the effects of arts appreciation or that of active arts participation. For example, a long research tradition has examined the so-called Mozart effect, in which Rauscher et al. (1993) observed that participants showed significantly higher spatial performance after listening to a Mozart sonata. Although, replications and meta-analyses have struggled to confirm these effects (e.g., Pietschnig et al., 2010). Second, arts can be taught as a stand-alone subject or be integrated into other subjects or the wider teaching approach. For example, arts integration has been found to be beneficial across art domains, ages and outcomes (e.g., Hardiman et al., 2014; Lee et al., 2015; Brown et al., 2018). However, the term arts integration is often interchangeably used for different approaches (Institute for Arts Integration and STEAM, 2019), varying from arts enrichment which uses the arts as a tool of engagement to using science, technology, engineering, the arts and math to guide students' critical thinking (STEAM; Perignat and Katz-Buonincontro, 2019). The distinction between different forms of engagement and degree of integration appears important to take account of the very different experiences and aims.

Third, a distinction can be made by the consideration of intrinsic (e.g., express communal meaning, McCarthy et al., 2004) vs. instrumental outcomes (e.g., academic outcomes). McCarthy and colleagues note that the former is rarely considered in the literature. In their review of arts and transfer into non-arts domains, Winner et al. (2013) critically discuss the sole focus on instrumental outcomes as these promote an evaluation of the arts as a means to an end rather than for their own sake. The Reviewing Education and the Arts Project (REAP) (Hetland and Winner, 2001; Winner and Hetland, 2001) included a series of meta-analyses to assess the relationship between arts engagement and academic outcomes. The results indicated that, despite some positive correlations, the only significant causal claims concerned the effects of music listening and music instruction on spatial reasoning (Hetland, 2000a,b) and of drama on verbal skills (Podlozny, 2000). Music training may also positively affect math outcomes (Vaughn, 2000); however, there was a too-large range of effects in a small number of studies to draw definitive conclusions. The same was true for dance, with some evidence found for effects on visual-spatial skills, but again based on a small number of studies (Keinänen et al., 2000). Evidence for the effects of the arts on creativity transfer could not be found (Moga et al., 2000). In 2013, a review published by the Organisation for Economic Co-operation and Development (OECD) updated and extended the findings of REAP (Winner et al., 2013) by including evaluations of behavioral and social outcomes. Evidence from quasi-experimental and experimental studies indicated social and behavioral benefits of drama, such as empathy, emotion regulation, and perspective-taking. Effects of music training were found on academic performance as well as intelligence, word decoding and phonological skills. There were also indications of a beneficial effect of music on language learning, but limited support for an effect on visual-spatial reasoning. Associations between drama or dance and creativity were apparent but based on only a few studies with small sample sizes, therefore limiting the ability to draw conclusions. A further series of reviews of quantitative and qualitative evaluation studies of arts in schools and the community by Jindal-Snape et al. (2014a,b, 2018) found similarly varied results concerning academic outcomes, with a tendency for effects to be more evident in music and multi-art contexts and more pronounced among pre-school children.

In general, the quantitative research to date provides mixed evidence regarding the effects of the arts on academic outcomes and largely remains on the correlational level. However, there are a few implications when interpreting these results. A difficulty when comparing results from different studies and programs is a lack of a common evaluation approach and programs' unique characteristics, contexts and quality (Ewing, 2010). Programs exhibit a substantial heterogeneity with respect to art domains, content, intensity, duration and delivery methods of arts classes or interventions, all across a small number of studies. For example, failing to distinguish between different forms of engagement with the arts (passive or active), how they are integrated into the school curriculum and whether they are offered in the form of a compulsory or extra-curricular activity adds many factors that may affect research results in different ways.

The current review aims to reduce some of the above heterogeneity by synthesizing effects from quasi-experimental or experimental pre-test post-test designs on the impact of arts education on a range of competencies. It seeks to evaluate programs which involve active involvement in the arts and teaching of the arts per se. Further, it aims to critically evaluate the strengths and limitations of the included studies. A wide range of outcomes will be included, and a framework of educational outcomes, the OECD's *Definition and Selection of Competencies* (DeSeCo; Rychen and Salganik, 2003) will be used to group outcomes into broader categories.

### A Categorization of Outcomes

We grouped outcomes into categories based on DeSeCo (Rychen and Salganik, 2003). The framework was the result of an international, interdisciplinary and collaborative effort led by Switzerland and supported by the OECD. Key competencies were identified in an extensive process bringing together empirical evidence, scholars and experts from different disciplines, including a final consolidation and revision phase. Providing a categorization of educational outcomes, the DeSeCo considers individuals' needs to lead a successful life and the needs of a functioning society. These competencies are summarized into three categories, namely *using tools interactively, interacting in socially heterogeneous groups*, and *acting autonomously*. First, *using tools interactively* encompasses key competencies relating to using the tools of language and symbols, technology, information and knowledge. Second, *interacting in socially heterogeneous groups* refers to the social skills needed to work collaboratively in a diverse and multicultural society, to be able to control one's emotions and to show empathy when relating to others and dealing with conflict. Third, *acting autonomously* encompasses the key competencies needed for an individual to make autonomous life choices and feel empowered to do so. Finally, an underlying feature that applies to all categories of competencies is *reflectiveness*, the ability to go beyond knowledge and skills, to assimilate, change and adapt. Reflectiveness therefore encompasses meta-cognitive skills as well as the cognitive skills essential for creative thinking.

Instrumental perspectives in evaluating arts education have frequently focused on outcomes relating to the DeSeCo category *using tools interactively* (e.g., by assessing transfer effects to non-arts subjects, linguistic or computational skills). However, given the more process focused, intrinsic experiences of the arts, one may expect stronger effects on the categories relating to the inter- and intra-individual outcomes (i.e., “soft” skills) than on transfer of learning to non-arts subjects (e.g., development of social bonds and empathy; McCarthy et al., 2004). While the DeSeCo categories were developed in consideration of competencies which are instrumental to a successful life, they may also contribute to the innate psychological needs for competence, relatedness and autonomy, which are the three key needs proposed in the motivational self-determination theory (Deci and Ryan, 1985). The DeSeCo framework was thus chosen as a tool to group a wide range of outcomes into categories and to enable an exploration of effects on similar and dissimilar outcomes.

### Research Questions

The current review seeks to systematically review competency-based outcomes and effect sizes in (quasi-) experimental studies of school-based arts education. Further, the review seeks to critically assess the qualities of the included studies in respect to limitations of (quasi-) experimental research in arts education. Research questions and exclusion and inclusion criteria are defined using the PICOS framework (participants, interventions, comparators, outcomes, and study design; Liberati et al., 2009). Participants are defined as children or adolescents from the general population attending formal compulsory education post pre-school. This review considers programs in which art is taught as a stand-alone subject with a minimum session duration of twenty minutes, as opposed to arts-integrated teaching within or in combination with other subjects, focusing on four art domains which are most frequently implemented in school curricula, namely the domains of music, drama, dance, and visual arts. Comparators include both active (treated) control groups participating in a non-arts or different arts program as well as untreated control groups following the standard curriculum. Studies reporting effect sizes on outcomes relating to the DeSeCo framework (Rychen and Salganik, 2003) are included. The included study designs are natural/quasi-experimental, or experimental with pre-test post-test designs. Based on the above PICOS definitions, the review seeks to answer the following exploratory research questions: (1) How effective is arts education with respect to outcomes in the DeSeCo categories (a) *using tools interactively*, (b) *interacting in socially heterogonous groups*, (c) *acting autonomously* and d) the concept of *reflectiveness*? (2) What are the methodological characteristics, strengths and limitations of the included studies?

## Methods

**Figure 1:**
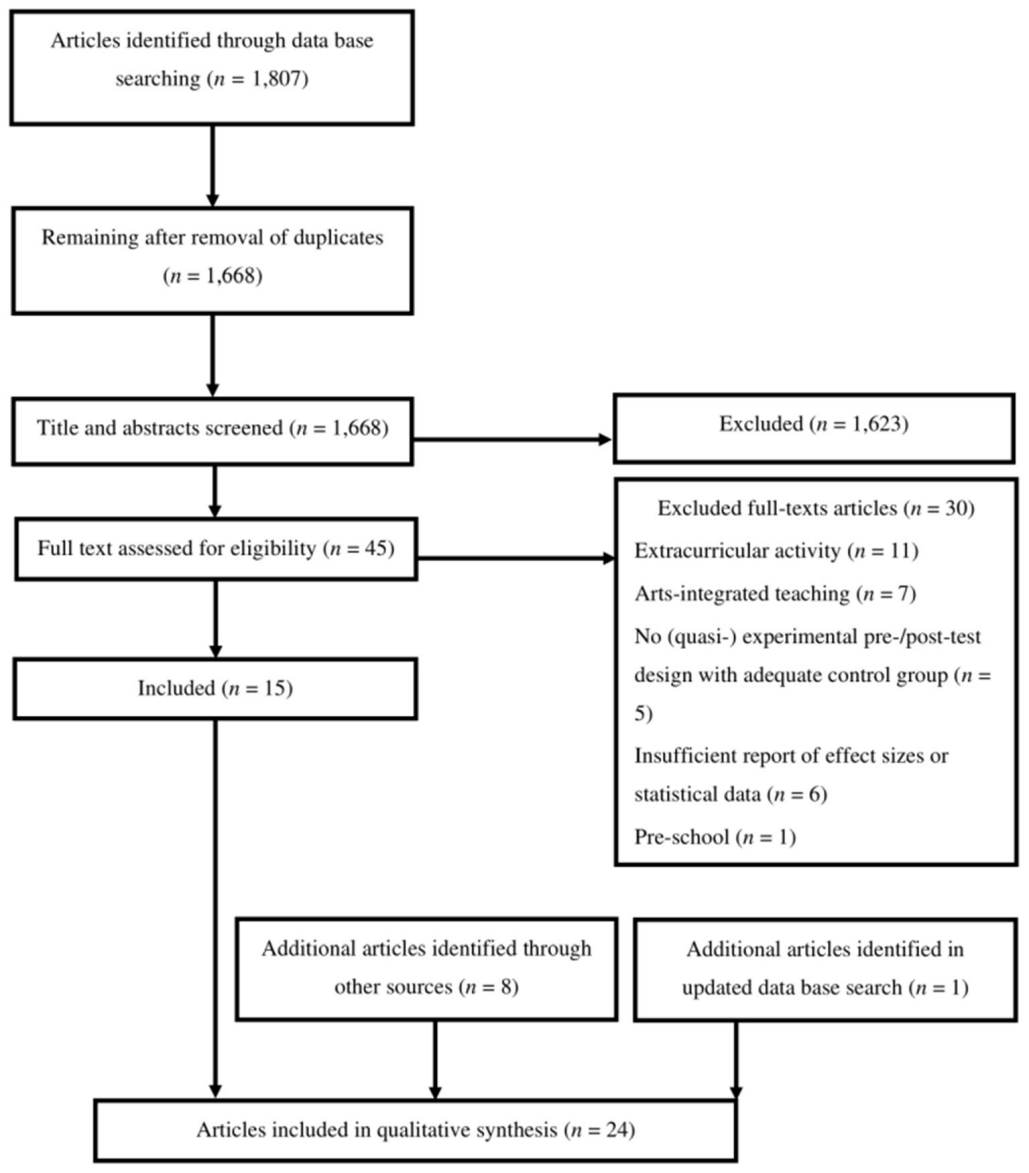
PRISMA flow diagram.

The initial literature search was conducted in March 2019, following the Preferred Reporting Items for Systematic Reviews and Meta-Analyses (PRISMA) guidelines (Moher et al., 2009). The databases PsycARTICLES, PsycINFO, Behavioral Science Collection, PSYNDEX, Education Source and ERIC were searched with search strings combining terms related to art domains, intervention evaluation, and thesaurus explorations of relevant outcome variables (Supplementary Table 1). Bibliographies of relevant meta-analyses and systematic literature reviews and further publications by authors with relevant studies were searched manually. The total number of search results was 1,807 from the databases and 13 from additional sources, leaving 1,681 search results after the removal of duplicates (Figure 1).

The first author screened the articles' titles, abstracts and full texts against the inclusion and exclusion criteria. Articles were included if they were research articles published in English or German in peer-reviewed journals, were evaluation studies of arts education for children and/or adolescents within the school curriculum, with outcome variables relating to the above DeSeCo categories (Rychen and Salganik, 2003) and if they used a quantitative or mixed methods approach with a natural/quasi-experimental or experimental pre- and post-test design. Excluded were studies reporting on extra-curricular programs (e.g., after-school clubs) or implemented in pre-schools, kindergarten, or post-school contexts (e.g., universities), that were integrated as therapeutic interventions or addressed specific populations (e.g., children with autism). Further, due to the risk of bias from other confounding effects, studies reporting on programs in which the arts were just one intervention component out of several (e.g., family counseling) were excluded. Finally, to take account of the problematic interpretation of results based on binary significance results (Siddaway et al., 2019), studies that reported neither effect sizes nor sufficient statistics allowing for the extraction and interpretation of effect sizes were also excluded. After title and abstract screening, the full texts of the 54 remaining articles were screened, and 23 articles reporting a total of 25 studies were retained. The search was updated in April 2020 (Supplementary Table 1) and one additional relevant study was identified that had been published after the first search. Ten percent of articles during the title and abstract screening and another ten percent of articles during the full text screening were double-screened by a second independent reviewer. Discrepancies between the two reviewers were resolved through discussion, leading to full agreement on the inclusion and exclusion of articles based on the pre-defined criteria.

Study characteristics relating to randomization, type of control group, sample size, participant age or grade, program intensity and duration, main outcome measures, results and effect sizes were extracted for each study and are summarized in Supplementary Table 2. Further, theorized mechanisms of change in the included studies were recorded. While the study inclusion was based on effect size estimates, the precision of effect size estimates is dependent on the sample size. To address concerns about the accuracy of effect size estimates from small samples, as well as a possible overestimation bias in eta-squared (Morris, 2008; Fritz et al., 2012; Lakens and Evers, 2014), additional confidence intervals and alternative effect sizes were calculated where possible (Lenhard and Lenhard, 2016; Uanhoro, 2017; Stangroom, 2019). Morris' (2008) recommendation on the use of effect sizes in pre-test post-test control group designs was applied where possible. These additional calculations were dependent on the availability of information in the papers regarding sample sizes, means and standard deviations of pre- and post-test scores. Furthermore, these calculations were limited to statistical designs that did not include covariates or adjustments for violations of test assumptions. Effect sizes were interpreted as small, medium and large according to Cohen's conventions (Cohen, 1988).

## Results

The following narrative summary of included studies is structured into two sections. The first section summarizes effects on outcomes relating to the DeSeCo categories *using tools interactively*, and the second section reports the outcomes on the “soft” skill outcomes in the categories *interacting in socially heterogonous groups, acting autonomously* and *reflectiveness*.

### Analysis of Studies With Outcomes Relating to the DeSeCo Categories *Using Tools Interactively*

#### Music Education

Twelve studies assessing outcomes of music education relating to the DeSeCo category *using tools interactively* fulfilled the inclusion criteria. Most used quasi-experimental designs, with the exception of two studies with experimental designs (Rickard et al., 2012, Study 2; Rabinowitch et al., 2013), and a treated control group was implemented in seven studies. Study characteristics were very heterogenous, with sample sizes varying between *n* = 28 and *n* = 345, and program intensity and duration varying from weekly 20-min classes over an 8-week period (Brodsky and Sulkin, 2011, Study 3) to 7 h per week over several years (e.g., Degé et al., 2011; Supplementary Table 2).

Two studies assessed arithmetic skills as an outcome of music education. A large effect size on arithmetic skills was extracted from one study of children receiving music composition classes (Bugos and Jacobs, 2012), and a small effect size on computation scores was found among children taking piano lessons (Costa-Giomi, 1999). Processing speed was assessed in three studies, revealing a medium effect of music composition training (Bugos and Jacobs, 2012) and trivial (Guo et al., 2018) to small effects of instrumental music training (Roden et al., 2014b). Working memory was assessed in five studies, and whilst there was some evidence for medium to large effects of music training on verbal or auditory components of working memory in three studies (Degé et al., 2011; Roden et al., 2012, 2014a), others had mixed results, with trivial and small effect sizes or even contrary results for the second year of intervention (Rickard et al., 2010; Guo et al., 2018). In contrast to auditory functions, visual functions, such as visual attention or memory, were generally not expected to be affected by musical education. The range of effect sizes, spanning from small with negative valence (Roden et al., 2014b) to null or small effects with positive valence (Rickard et al., 2010; Bugos and Jacobs, 2012; Roden et al., 2012) and medium positive effect sizes Degé et al. (2011), may support the expected absence of effects of music education on visual functions. Furthermore, the effects of music education on vocabulary and verbal learning variables ranged from small effect sizes with negative valence to small effect sizes with positive valence (Rickard et al., 2010; Bugos and Jacobs, 2012; Guo et al., 2018), with the exception of two medium-sized effects on language skills (Costa-Giomi, 1999) and verbal learning (Rickard et al., 2010). Brodsky and Sulkin (2011, Study 3) reported a large effect size on a composite score of dictation content and technical aspects of handwriting. No effects of music education on overall intelligence were found (Degé et al., 2011).

#### Drama Education

One experimental study with an untreated control group evaluated the effect of a 2-hour long weekly drama class with a total duration of 12 weeks on measures of intelligence (Köksal Akyol, 2018). In the sample of 46 first grade children, a medium effect of the drama intervention on the verbal-linguistic intelligence subscale of the Turkish adaptation of the Teele Inventory for Multiple Intelligences (TIMI; adapted by Göǧebakan, 2003, as cited in Köksal Akyol, 2018) did not reach significance. No effects were found on the mathematical-logical or visual-spatial subscales.

#### Comparisons Between Different Art Domains

Four evaluation studies included in this review compared the effects of an arts program to that of another arts program in a different domain (Rickard et al., 2012; François et al., 2013; Hogenes et al., 2016). Two studies were experimental, and sample sizes ranged from *n* = 24 to *n* = 133.

Comparing the verbal learning and verbal intelligence over time of students who received an increased frequency of music or drama lessons (Rickard et al., 2012, Study 1) revealed no differences in verbal learning and delayed recall. However, a small effect size corresponding to greater improvement among music students was found for measures of immediate recall. When comparing a group of music students to a painting group, music training affected speech segmentation processes with a large effect size (François et al., 2013). Regarding non-verbal intelligence, a large extracted effect size indicated that students receiving increased music compared to increased drama classes exhibited greater improvement (Rickard et al., 2012, Study 1). Finally, Hogenes et al. (2016) compared the effects of a music composition to a music performance intervention. A medium effect size for the between-group differences on post-test reading comprehension was due to a larger decrease among students in the music performance group when controlling for pre-test scores.

#### Proposed Mechanisms of Change

Several mechanisms to explain effects of the arts on non-arts outcomes were proposed by the authors of the included studies. For example, it was suggested that the simultaneous processing of multiple sensory cues required when playing music can explain working memory enhancement, specifically enhancement of auditory components, including verbal memory (e.g., componential model of working memory, Baddeley, 2010; Roden et al., 2014a). Others propose that improved attention and mental processing speed due to music engagement may cause effects on academic performance (Roden et al., 2014b).

Other indirect pathways are proposed by applying motivational, social learning and evolutionary theories to the context of arts learning. Arts engagement may offer opportunities to experience flow (Csikszentmihalyi, 1975) and therefore improved attention and concentration (Bugos and Jacobs, 2012). Other pathways involving motivational learning processes have been suggested with respect to collaborative learning in the arts (e.g., Vygotsky, 1977). An evolutionary perspective was applied to the domain of childlore and handclapping songs (Brodsky and Sulkin, 2011, Study 3). The latter was described as a naturally occurring activity in 6–9-year-old children, with evidence of its existence dating back to 2000 BC. Combining sensory-motor, social and verbal/linguistic elements, the study's authors propose an evolutionary developmental purpose predicted to affect development in non-arts domains such as motor and cognitive development (Brodsky and Sulkin, 2011, Study 3).

### Analysis of Studies With Outcomes Relating to the DeSeCo Categories *Interacting in Socially Heterogonous Groups, Acting Autonomously* and *Reflectiveness*

#### Music Education

Five evaluation studies of musical programs included in this review addressed outcomes relating to the DeSeCo categories *interacting in socially heterogonous groups* or *acting autonomously*. Two studies used an experimental design and three studies a treated control group, with sample sizes varying between *n* = 24 and *n* = 84. The intensity and duration of interventions ranged from weekly 30–60-min classes over a 3-month to a 3-year period.

Effect sizes regarding social skills and behavior were extracted from four studies (Rickard et al., 2012, study 2; Rabinowitch et al., 2013; Schellenberg et al., 2015; Roden et al., 2016). Whilst Roden and colleagues found that extended music training buffered against increases in aggressive behavior observed in the control group, an effect of large size, the opposite trend was observed for self-reported aggression in the study by Rickard and colleagues. Rabinowitch and colleagues found that an interactive musical intervention improved empathy in the music group compared to the control group, an effect of medium size. For a subgroup with lower social skills at baseline, Schellenberg and colleagues reported large effects of the group × time interaction on sympathy and prosocial behavior due to significant increases in the music group. Regarding intra-personal outcomes, two studies assessed effects of music education on self-esteem (Costa-Giomi, 1999; Rickard et al., 2012, Study 2), but neither study reported effect sizes. Costa-Giomi (1999) reported a significant effect on self-esteem among children receiving piano lessons and computations revealed a small effect. However, Rickard et al. (2012) did not report significant group × time interactions when comparing three groups of music, drama and control students over time.

#### Drama Education

Five studies assessed drama interventions' effects on social competency outcomes (Walsh-Bowers, 1992; Walsh-Bowers and Basso, 1999; Rickard et al., 2012; Köksal Akyol, 2018). Four of these applied a non-treated control group and two randomized group allocation with sample sizes varying from *n* = 44 to *n* = 104. Program intensity and duration ranged from one 40-min to five 60-min classes per week over periods of 12–15 weeks.

Köksal Akyol (2018) reported no experimental effects on intrapersonal and interpersonal intelligence measures. In fact, effect sizes calculated from the data provided in the article suggest a small effect on interpersonal intelligence in the opposite direction as expected. Three studies conducted by Walsh-Bowers and colleagues (Walsh-Bowers, 1992; Walsh-Bowers and Basso, 1999) assessing social skill development in adolescents in a drama program showed mixed results. There were generally no effects on self-rating scales of confidence in social situations, except for one small effect on self-rated cooperation (Walsh-Bowers and Basso, 1999; Study 1). Intervention effects on teacher ratings of social skills differed widely between studies, ranging from medium and large effect sizes in support of the drama program to large effects in the opposite direction, and intervention effects on parents' ratings of social skills ranged from small to large across studies in support of the intervention. As with music students, self-rated aggression scores among drama students seemed to increase in comparison to a non-treatment control group over time (Rickard et al., 2012, Study 2). However, the group × time interaction was only marginally significant, and the effect was only interpreted based on descriptive statistics of the three groups at pre- and post-test.

One experimental study with a treated control group assessed non-verbal divergent thinking after a single drama improvisation session in a sample of 34 ten- and eleven-year-old sixth grade schoolchildren (Sowden et al., 2015a). Large intervention effect sizes for the originality and elaboration measures from the Incomplete Figures Tasks of the Torrance Tests of Creative Thinking (Torrance, 1974, as cited in Sowden et al., 2015a) were found. The effect size for abstractness was non-significant and its size was not reported.

#### Dance Education

Two studies evaluated the social outcomes of the weekly 90-min dance intervention TanzZeit [time to dance], taught over a period of one or two semesters in quasi-experimental designs using untreated control groups (Zander et al., 2014; Kreutzmann et al., 2018). The studies employed sizable samples (*n* = 606 and *n* = 361).

Both studies reported significant treatment effects on variables assessing affective and/or collaborative social networks, measured by asking students to indicate how much they liked or liked collaborating with each classmate, and their feelings of social belonging. Small effects of the dance intervention on affective networks were found in one study (Kreutzmann et al., 2018), but these were trivial in the study by Zander et al. (2014). Kreutzmann and colleagues also reported evidence for affective social networks mediating the relationship between the dance intervention and sense of social belonging. However, effect sizes for direct and indirect paths to social belonging remained on the trivial level, and sensitivity analysis revealed that their sizes were dependent on the contrast codes employed. For collaborative networks, Zander and colleagues reported small intervention effects in the subsample of boys, and large intervention effects for boys' opposite-sex nominations. These effects were not observed in the subsample of female participants.

One experimental study assessed the effects of a 20-min folk dance physical activity on creativity outcomes among Indian children (Bollimbala et al., 2019). A sample of 34 sixth and seventh grade children were randomly assigned to a 20-min class or a sedentary control group. Pre- and post-tests of divergent and convergent thinking revealed small experimental effects on convergent thinking only, and a small effect in the opposite direction was extracted on flexibility in divergent thinking across the entire sample. However, in the subgroup of children with normal body mass index (BMI), as opposed to the other half of the sample with low BMI, small experimental effects were revealed on all dimensions of the divergent and convergent thinking measures.

#### Visual Arts Education

One evaluation study assessed the outcomes of two visual arts programs on self-efficacy, self-concept, internal and external success attributions, worldviews and self-reported creativity (Catterall and Peppler, 2007). A total of 179 nine to 10-year-old third grade children were assessed in a quasi-experimental design with untreated control groups after 20–30 weeks of weekly 60- to 90-min classes. Significant self-efficacy gains were observed among children in the visual arts groups, an effect of medium size in relation to the changes in the control groups. Differences between groups and over time on self-concept, internal and external success attributions and worldviews were not significant and effect sizes not retrievable. Self-reported creativity measures were based on the dimensions of the Torrance Test of Creative Thinking (Abedi, 2002). A large treatment effect was extracted for the creativity dimension of originality. No effect sizes could be extracted for the non-significant effects on the creativity dimensions of fluency, flexibility and elaboration.

#### Comparisons Between Different Art Domains

One experimental (Freeman et al., 2003) and one quasi-experimental study (Rickard et al., 2012, Study 1) comparing the effects of music and drama interventions allowed for the extraction of relevant effect sizes on measures of self-image, self-concepts, self-esteem, and attitudes. Sample sizes ranged from *n* = 111 to *n* = 185.

Freeman et al. (2003) reported no significant post-test differences between music and drama groups. However, small effects according to Morris (2008) calculated on a subsample with both pre- and post-measures indicate that drama students exhibited greater improvement in self-reported self-image and academic self-concept, and music students' greater improvement in social self-concept. No differences were found in teacher ratings of social skills and problem behavior. Rickard et al. (2012) did not report significant group × time interaction effects on the respective outcomes. However, small effect sizes according to Morris (2008) indicate stronger effects of drama education on academic self-esteem, general attitudes and attitudes toward social integration compared to the music group. Similarly, with medium effect sizes, drama had stronger effects on attitudes toward teaching and status among peers and medium to large effect sizes on engagement and motivation in arts class.

#### Proposed Mechanisms of Change

It has been proposed that music engagement may promote empathic processes and social cohesion through the experience of rhythmic and movement cohesion (Rabinowitch et al., 2013; Schellenberg et al., 2015), which can be explained from an evolutionary perspective (Huron, 2001; Tarr et al., 2014). Similar mechanisms have been proposed for the effects of dance interventions on social outcome variables (Zander et al., 2014; Kreutzmann et al., 2018), with the additional aspect of physical proximity and opportunity for contact being a possible catalyst for friendships (theory of social impact, Latané et al., 1995). According to Kreutzmann and colleagues, dance choreography also creates an interdependency among participants which may foster these social variables (social interdependence theory, Johnson and Johnson, 2009). The drama intervention by Walsh and colleagues (structured fantasy approach, Walsh et al., 1991) was derived from social development theory and the role of play for becoming competent in social situations (Vygotsky, 1967). Similarly, Freeman et al. (2003) propose that elements of social skills trainings are integral to creative drama and, like Catterall and Peppler (2007), propose that mastery experiences from the creative classroom can transfer to generalized self-efficacy, as theorized in Bandura's social cognitive theory (Bandura, 1986).

Catterall and Peppler, 2007 assessed creativity outcomes of visual arts programs and propose that visual arts programs provide the learning environment to engage in metacognitive activities, which are central in Bruner's (1966) constructivist model, as well as active and collaborative learning (situated learning, Lave and Wenger, 1991; distributed learning, Bransford and Schwartz, 1999). Sowden et al. (2015a) applied the dual-process theory of creative thinking (Sowden et al., 2015b) to identify the creativity processes, namely divergent thinking, they expected to be specifically affected by an improvisation class.

Four studies reported mixed method approaches by incorporating observational and interview data (Walsh-Bowers, 1992; Walsh-Bowers and Basso, 1999; Catterall and Peppler, 2007) to understand student's responses to the intervention, their engagement and processes of development.

### Risk of Bias

Several common risks of bias were identified across studies. Just over a quarter of the included studies (26.9%) fully randomized participants into groups, thereby minimizing systematic variation not caused by the experimental manipulation. Just over half of the studies (51.9%) included a treated control group. Such a comparison group is important to distinguish a treatment effect from a behavior change merely due to the novelty or increased attention involved in participating in a new program, similar to a placebo effect (Winner et al., 2013). Less than a third of studies evaluated intervention durations of more than a year (30.8%), while 15.4% of studies evaluated one-off classes or interventions with a maximum 10 weeks duration.

Only 7.4% of included studies recruited a sample size based on an *a priori* power analysis. Furthermore, 88.5% of studies employed a sample size smaller than *n* = 200, which would be sample size required to detect a small time × group interaction effect in a 2 × 2 ANOVA (calculation in G^*^Power 3; Faul et al., 2007; *f* = 0.10, α = 0.05, 1–β = 0.80, correlation among repeated measures = 0.5, non-sphericity correction = 1). Insufficient statistical power has implications for the reliability of significance tests and effect size estimates. Information on participant attrition could be extracted from 12 studies, of which one study had low attrition (below 5%), five studies medium (5–20%) and six studies large attrition (more than 20%; attrition classification according to Schulz and Grimes, 2002). Just over two-thirds of the studies provided reliability information for the instruments used. Of these, half (nine studies) reported acceptable reliability values (≥0.70) for all employed instruments.

## Discussion

### Answers to the Research Questions

A systematic review of the literature was conducted in order to answer the following questions: (1) How effective is arts education with respect to outcomes in the DeSeCo categories (a) *using tools interactively*, (b) *interacting in socially heterogonous groups*, (c) *acting autonomously* and (d) the concept of *reflectiveness*? (2) What are the methodological characteristics and limitations of the included studies? Regarding the outcome category *using tools interactively*, most eligible studies reported on outcomes of music education. Wide ranges of effect sizes were found across outcome variables. This variation may be due to generally small sample sizes and unreliable effect size point estimates, as indicated by the large confidence intervals. There was some evidence of the potential benefit of music training on processing speed based on a few studies with small to medium effect sizes. However, no such effects were found in another study (Guo et al., 2018). Music students outperformed painting students with a large effect size difference in speech segmentation, as assessed by test performance and event-related potentials (François et al., 2013). Inconsistent and trivial effects of music education were observed on vocabulary, visual memory, visual performance, and non-verbal intelligence. There was some support for effects of music training on arithmetic abilities, but this was only based on a few studies and effect sizes ranged from small to large. No effect of music training was found on global or verbal intelligence scores; however, a large effect emerged for non-verbal intelligence compared to a painting group. These mixed findings stand in contrast to previous studies finding that music education increases intelligence. For example, in an evaluation of a free community music program, Schellenberg (2004) pre-assessed a sample of 6-year-old children before entering first grade. At 1-year follow-up, small to medium-sized effects of music training on intelligence were found when compared to children with drama or no treatment. However, the effects of music education on cognitive outcomes may be moderated by age. Developmental gains and plasticity in younger children could explain differences by age in some cognitive effects of arts education.

Three studies assessed creativity outcomes of drama improvisation, visual arts or dance classes. Large effects were found for divergent thinking, mostly the originality dimension, after drama or visual arts classes. However, effects on divergent thinking were not as clear in an Indian sample of children receiving a twenty-minute folk dance class, where the presence of an effect seemed to be moderated by the children's BMI (Bollimbala et al., 2019). It is important to note that two of these studies assessed the children's performance on creativity tests immediately after a single one-off class. Therefore, the effect may represent a momentary state of mind. On the other hand, longer-term effects of regular visual arts classes were only measured using a self-report creativity measure, and comparisons across instruments should be made with caution (Catterall and Peppler, 2007). Based on the included studies, conclusions about the sustainability of creativity over time and differential effects of longer-term versus one-off classes cannot be made.

Regarding the outcome categories *interacting in socially heterogonous groups* and *acting autonomously*, a small number of studies were eligible for inclusion in this review. These examined a range of different outcomes with often inconclusive or even contrasting results. For interpersonal outcomes, positive results with medium and large effects were reported for music training on the related concepts of empathy and sympathy as well as prosocial behavior. However, these were based on only a few studies. Furthermore, when looking at the effect of music education on reducing unfavorable behavior, such as aggression, the results were mixed across studies. Likewise, the evidence for effects of dance and drama on interpersonal outcomes was uncertain due to a small number of studies and contrasting or trivial effect sizes. Dance effects on some interpersonal outcomes may be moderated by gender, an observation that calls for more research applying moderation analyses. Finally, with respect to intrapersonal outcomes, the suggested positive effects of music on self-esteem and of visual arts on self-efficacy were based only on single studies, which precludes a general conclusion.

The limited support for social-emotional outcomes of arts education in this review contradicts a plausible expectation of such benefits, for instance due to the increased opportunities for social interactions and collaboration which are characteristic of performing arts. For example, previous research found some evidence for social and emotional benefits of drama on empathy, perspective taking and emotional regulation, in line with such expectations (Winner et al., 2013). A scoping review of the role of the arts for health and well-being published by the World Health Organization (Fancourt and Finn, 2019) delivered further evidence for beneficial effects of the arts on social cohesion among different populations in the community and particularly wide-ranging social-emotional benefits for socio-economically disadvantaged groups. However, the small number of included studies and a number of limitations of these studies discussed below call for caution to draw conclusions. Further, while this review has focused solely on the class level, more subtle individual changes may be missed. The following discussion of the second research question will also debate the choice of outcomes and methods for their assessment.

The second aim of the review was to answer the question: What are the methodological characteristics, strengths and limitations of the included studies? The review's inclusion criteria focused on quasi- and experimental studies with pre-test post-test designs and effect size estimates. While quasi-experimental studies are limited in their ability to provide causal evidence, experimental studies are commonly considered the “gold-standard” to demonstrate causal impact. Pre- and post-measures allow for the control of changes over time and effect sizes and confidence intervals enable an estimation of the size and certainty of effects. However, the restrictive inclusion criteria resulted in a small number of included studies, of which only 27% used randomization into intervention and control groups. The scarcity of the so-called “gold standard” research method in the field may be partly due to practical considerations and financial limitations. Further, it may be a reflection of a controversy between the need of rigorous evaluations, what scientific methods are appropriate for evaluations and the concern that experimental methods may be too reductionist to be suitable for more holistic and complex experiences and impacts of the arts. As discussed in the context of complementary medicine by Mason et al. (2002), these concerns are not unreasonable and need to be acknowledged in the design of rigorous evaluation studies.

For example, programs evaluated in (quasi-) experimental designs tend to be shorter in duration and/or follow-up compared to correlational research and may not provide long enough exposure and follow-up to uncover meaningful effects (Catterall, 2002; McCarthy et al., 2004). For example, this review also included studies which only evaluated one-off classes or very short educational programs with short retest intervals. It has been suggested that a minimum intervention duration of one year is required to determine if a transfer effect occurred (Moga et al., 2000). Only eight of the included studies met this criterion. Results from short programs therefore need to be interpreted with caution.

Second, sample sizes tend to be smaller in (quasi-) experimental designs. This results in small statistical power and, alongside short follow-up periods and variations in individual experiences, non-significant cohort effects. A majority of the included studies had small sample sizes and would have been insufficiently powered to discover small effects.

Third, arts programs are more difficult to standardize and evaluation research needs to take the impact of the practitioner and participant relationship and adaptations in delivery into account. According to Ewing (2010), stronger interventions are the ones that can be adapted to the participants and contexts which makes comparison across interventions more difficult.

Finally, outcomes need to be broad enough or appropriate for the respective program. As illustrated in the introduction, the choice of exclusively instrumental outcomes has been critiqued as it disregards the intrinsic outcomes of the arts. While the DeSeCo framework selected for the categorization of outcomes in this review adopts a wider perspective and arguably includes intrinsic processes in intra- and interpersonal level outcomes, the limitations of this perspective will be discussed below. In addition, qualitative research can be illuminating to understand relevant outcomes and complex real-world experiences. As proposed by Paluck (2010), field experiments do not need to be and should not be exclusively quantitative but can be integrated with qualitative methods. The integration of qualitative data allows to shed light on pathways and outcomes and can strengthen the conclusions from the quantitative data. For example, in an evaluation of the impact of Creative Partnerships (CP) on children's wellbeing, the quantitative survey study with CP and control schools was complemented with qualitative case studies (McLellan et al., 2012b). While the former did not find statistically significant effects between the schools, the latter illuminated mechanisms in high-wellbeing schools which can be fostered through arts integration and differences between the CP and control school (Galton and Page, 2014; McLellan and Steward, 2014). In another example in one of the here included studies, Catterall and Peppler (2007) conducted observations of the visual arts and home classrooms in both intervention and control schools. The results of the observations complemented their survey-based data by suggesting how the arts classes may have produced the observed effects. They observed higher student engagement in arts classes, more positive relations between peers and with adults, a change in how teachers viewed students and significant improvements in the integration of two case study students. Thus, the insights from the qualitative data can inform the design of future studies to test processes and help theory building.

Hence, the integration of qualitative methods into field experiments seems to be a promising approach. As the above examples illustrate, the combination of observation of classroom sessions and interviews of students and teachers within a field experiment enables a fuller elaboration of the mechanisms and complexities in arts education. First, observations allow the study of processes in the classroom, the in-depth analysis of program characteristics and nuances in the delivery, engagement and relationships in the classroom. Thus, the nature of experiences and specific ingredients of programs can be more fully understood and compared. Second, interviews with students and teachers in intervention and control groups can help to understand the complex experiences in and outside the respective classroom. For example, in an evaluation of the impact of a music orchestral program on collaborative skills, students may be asked about how they like collaborative work and to comment on experiences that created their like or dislike. Further, they could be asked questions about their collaborative relationships with peers at school and to give examples of when they feel collaboration went well or not so well. Interviews also enable a wider exploration of outcomes than what has been pre-specified in the quantitative data collection method. Therefore, rich data from qualitative research can help to support quantitative (experimental) research by illuminating important program characteristics, experiences, pathways and outcomes which are difficult to uncover in exclusively quantitative group level analysis.

As discussed above, there is a controversy about the appropriateness of experimental designs in the evaluation of the arts and holistic interventions. It may be fruitful to shift the focus of the discussion away from the experimental design and toward the limitations of associated shortcomings, such as the concern of being reductionist. Whilst a critical discussion of the “gold standard” is reasonable, the experimental design is not incompatible with some of the solutions discussed above, including qualitative research.

### Strengths and Limitations

One of the key objectives of the current review was to provide an overview of (quasi-)experimental evaluation studies of school-based arts education with pre-test post-test designs in order to identify what can be concluded from the evidence so far. Given the small sample sizes prevalent in this research field, this review focused on available and retrievable effect size estimates and confidence intervals. While the focus on effect sizes has significantly reduced the number of included studies, it allowed for a non-binary interpretation of the results, and the application of caution in consideration of the (un)certainty of evidence. From the narrative synthesis of the included studies, the review was able to summarize the limitations and gaps in the (quasi-)experimental research.

Yet, this review has also some limitations that should be considered. The formulation of the search strings may have missed relevant studies. However, by screening previous reviews and additional publications by the authors of included papers, potential bias due to the search strategy was minimized as much as possible.

Furthermore, the selected inclusion and exclusion criteria pose some limitations with implications for the interpretation of the results. First, the small number of quantitative evaluation studies in the field made it impractical to further narrow the selection down based on additional, albeit important design (e.g., randomization, sample size, follow-up duration) or context characteristics (e.g., age group). Consequently, the heterogeneity of the included studies with regard to these aspects limited the conclusions that could be drawn across them. Additionally, the criteria generated only a very small number of eligible studies for most art domains except music. These were insufficient to answer the research questions in this review, highlighting the need for further experimental research with sufficient sample sizes in the field, especially in the domains of drama, dance and visual arts. Second, the limitation to studies in peer-reviewed journals meant that the review excluded unpublished literature. Risk of publication bias cannot be ruled out. Third, while the exclusion of after-school clubs or programs outside of the school context attempted to minimize biases due to accessibility and self-selection, not all included studies delivered programs within the mandatory curriculum and thus, some self-selection biases cannot be excluded. Fourth, the choice of the DeSeCo framework and exclusion of studies in therapeutic setting will have excluded intrinsic outcomes of arts education not covered by the criteria. The results of this review should thus not be understood as a generalizable claim about the impact of the arts in general and rather be considered as an overview and critical analysis of the limitations of the available (quasi-)experimental research in terms of its outcomes and methodological characteristics.

The current review focused on universal effects on the group level, whilst the elaboration of moderators was not an objective. However, it is important to note that some effects may be stronger for different subgroups. For example, age has been shown to be related to intervention effect sizes, with greater effects for pre-school interventions compared to school-based interventions (e.g., Campbell and Ramey, 1994; Ulubey, 2018). Similarly, other characteristics such as gender should be part of future moderator considerations. A few studies included in this review assessed gender effects (e.g., Zander et al., 2014), (mal-)nutrition (Bollimbala et al., 2019) and baseline levels of the outcome variable as moderators (Schellenberg et al., 2015).

## Conclusion

The present review systematically searched and summarized the evidence on the effects of arts education (namely music, drama, dance, and visual arts) from quasi-experimental and experimental studies and critically discussed the evidence to date. Most included studies evaluated music programs, while the evidence base for drama, dance and visual arts is sparse. Largely, the number of studies and methodological characteristics did not justify definite conclusions. Whereas experimental designs are considered the “gold standard” to demonstrate causal impact, the present review has demonstrated that the “gold standard” is often associated with a range of methodological limitations which create uncertainty in the conclusions. To support hypothesized mechanisms and theory building, the inclusion of qualitative methods into field experiments seems a promising method as illustrated by a few examples.

## Data Availability Statement

The original contributions presented in the study are included in the article/Supplementary Material, further inquiries can be directed to the corresponding author/s.

## Author Contributions

AR and VS designed the review. VS was responsible for writing the initial and final version of the paper, conducting the systematic searches, and completing data extraction. AR supervised the study and contributed to the discussion and during the paper writing process. Both authors contributed to and approved the final manuscript for publication.

## Conflict of Interest

The authors declare that the research was conducted in the absence of any commercial or financial relationships that could be construed as a potential conflict of interest.
