# Supplementary material for: Arts in Education: A Systematic Review of Competency Outcomes in Quasi-Experimental and Experimental Studies
Source: Front Psychol. 2021 Apr 15;12:623935. doi: 10.3389/fpsyg.2021.623935 (PMC8082240; doi:10.3389/fpsyg.2021.623935)
Supplement: Supplementary file 1 [file Table_1.pdf]

Supplementary Material

Supplementary Table 1. Summarized search strategy.

Data base: PsychARTICLES, PsychINFO, Behavioral Science Collection, PSYNDEX, Education Source, ERIC	
	DE ("Art" OR "Crafts" OR "Drawing" OR "Painting (Art)" OR "Photographic Art" OR "Sculpturing" OR "Arts" OR "Artists" OR "Dance" OR "Improvisation" OR "Music" OR "Theatre" OR “Musical Instruments” OR “Drama” OR “Singing” OR “performing arts” OR “visual arts” OR "Kunst" OR "Handwerk" OR "Zeichnen" OR "Malerei (Kunst)" OR "Photogra*ieren" OR "Bildhauerei" OR "Künste" OR "Künstler" OR "Tanz" OR "Improvisation" OR "Musik" OR "Theater" OR “Musikinstrumente” OR “Drama” OR “Singen” OR “darstellende Künste”)
AND	DE ("Social Behavior" OR "Aggressive Behavior" OR "Involvement" OR "Prosocial Behavior" OR "Respect" OR "Responsibility" OR "Social Interaction" OR "Social Networks" OR "Social Skills" OR "Socioemotional Functioning" OR "Achievement" OR "Communication Skills" OR "Competence" OR "Learning Ability" OR "Nonverbal Ability" OR "Reading Skills" OR "Self-Care Skills" OR "Creativity" OR "Intelligence" OR "Performance" OR "Social Skills Training" OR "Social Integration" OR "School Integration" OR "Conflict Resolution" OR "Interpersonal Interaction" OR "Interpersonal Relationships" OR "Nonviolence" OR "Social Learning" OR "Imitation (Learning)" OR "Social Emotional Learning" OR "Psychosocial Outcomes" OR "Psychosocial Development" OR "Self-Perception" OR "Interoception" OR "Self-Efficacy" OR "Self-Knowledge" OR "Academic Self Concept" OR "Self-Concept" OR "Self-Confidence" OR "Self-Congruence" OR "Self-Regard" OR "Sense of Coherence" OR "Self-Esteem" OR "Belonging" OR "Caring Behaviors" OR "Intersubjectivity" OR "Empathy" OR “Bullying” OR "Cooperation" OR "Emotional Regulation" OR "Emotional Control" OR "Anger Control" OR "Self-Control" OR "Delay of Gratification" OR "Self-Regulation" OR "Collaboration" OR "Participation" OR "Peer Relations" OR "Role Taking" OR "Role Perception" OR "Cognitive Development" OR "Psychological Development" OR "Intellectual Development" OR "Language Development" OR "Brain Development" OR "Cognition" OR "Speech Development" OR "Theory of Mind" OR "Trust (Social Behavior)" OR "Academic Achievement" OR "Mathematics Achievement" OR "Reading Achievement" OR "Science Achievement" OR "Academic Achievement Motivation" OR "Engineering" OR "STEM" OR "Mathematics" OR "Sciences" OR "Biology" OR "Chemistry" OR "Computer Science" OR "Geography" OR "Information Science" OR "Medical Sciences" OR "Neurosciences" OR "Physics" OR "Mathematical Ability" OR "Cognitive Ability" OR "Brain Training" OR "Reading Ability"

	OR "Spatial Ability" OR "Verbal Ability" OR "Science Education" OR "Technology" OR "Verbal Fluency" OR "Academic Aptitude" OR "Language Proficiency" OR "Oral Communication" OR "Verbal Communication" OR "Verbal Memory" OR "Writing Skills" OR "Literacy" OR "Autonomy" OR "Empowerment" OR "Independence (Personality)" OR "Internal External Locus of Control" OR "Self-Determination" OR "Awareness" OR "Attention" OR "Goal Setting" OR "Aspirations" OR "Aspiration Level" OR "Educational Aspirations" OR "Goal Orientation" OR "Motivation" OR "Achievement Motivation" OR "Agency" OR "Goals" OR "Intrinsic Motivation" OR "Needs" OR "Social Motivation" OR "Self-Actualization" OR "Affective Education" OR "Ability" OR "Ability Level" OR "School Attendance" OR "School Refusal" OR "Truancy" OR "Student Attrition" OR "School Leavers" OR "Divergent Thinking" OR "Innovation" OR "Emotional Development" OR "Emotional Intelligence" OR "Health" OR "Well Being" OR "Self-Regulated Learning" OR "Transfer (Learning)" OR "Persistence" OR "Self-Monitoring (Personality)" OR "Resilience (Psychological)" OR "Adaptability (Personality)" OR "Coping Behavior" OR "Emotional Adjustment" OR "Emotional Stability" OR "Protective Factors" OR "Psychological Endurance" OR "Psychological Stress" OR "Soziales Verhalten" OR “Sozialverhalten” OR "Aggressionsverhalten" OR "Engagement" OR "Prosoziales Verhalten" OR "Respekt" OR "Verantwortung" OR "Soziale Interaktion" OR "Soziale Netzwerke" OR "Soziale Fertigkeiten" OR "Leistung" OR "Kommunikationsfertigkeiten" OR "Kompetenz" OR "Lernfähigkeit" OR "Nonverbale Fähigkeit" OR "Lesekompetenz" OR "Selbsthilfevermögen" OR "Kreativität" OR "Intelligenz" OR "Training sozialer Fertigkeiten" OR "Soziale Integration" OR "Schulische Integration" OR "Konfliktlösung" OR "Interpersonale Interaktion" OR "Interpersonale Beziehungen" OR "Gewaltlosigkeit" OR "Soziales Lernen" OR "Imitationslernen" OR "Sozialemotionales Lernen" OR "Psychosoziale Ergebnisse" OR "Psychosoziale Entwicklung" OR "Selbstwahrnehmung" OR "Selbstwirksamkeit" OR "Akademisches Selbstkonzept" OR "Selbstkonzept" OR "Selbstbewusstsein" OR "Selbstkongruenz" OR "Selbstachtung " OR "Kohärenzgefühl" OR "Selbstwertgefühl " OR "Zugehörigkeitsgefühl" OR "Intersubjektivität" OR "Empathie" OR “Mobbing” OR "Kooperation" OR "Emotionale Regulation" OR "Emotionale Kontrolle" OR "Ärgerkontrolle" OR "Selbstkontrolle" OR "Belohnungsaufschub" OR "Selbstregulation" OR "Kollaboration" OR "Partizipation" OR "Peer-Beziehungen" OR "Rollenübernahme" OR "Rollenwahrnehmung" OR "Kognitive Entwicklung" OR "Psychologische Entwicklung” OR "Intellektuelle Entwicklung" OR "Sprachentwicklung" OR "Gehirnentwicklung" OR "Kognition" OR "Sprechentwicklung" OR "Theory of Mind" OR "Vertrauen" OR "Akademische Leistung" OR "Mathematikleistung" OR "Leseleistung" OR "Naturwissenschaftliche Leistungen" OR "Leistungsmotivation" OR "Leistungsmotivation (Schule und Hochschule)” OR "Ingenieurwesen" OR "MINT-Fächer" OR "Mathematik" OR "Naturwissenschaften" OR "Biologie" OR "Chemie" OR "Computerwissenschaft" OR "Geografie" OR "Informationswissenschaft" OR "Medizinwissenschaft" OR "Neurowissenschaft" OR "Physik" OR "Mathematische Fähigkeit" OR "Kognitive Fähigkeit" OR "Kognitives Training" OR "Lesefähigkeit" OR "Räumliches Vorstellungsvermögen" OR "Verbale Fähigkeiten" OR "Naturwissenschaftlicher Unterricht" OR "Technologie" OR "Sprachgewandtheit" OR "Fähigkeit (Schule und Hochschule)" OR "Sprachtüchtigkeit (Zweitsprache)" OR "Mündliche Kommunikation" OR "Verbale Kommunikation" OR "Verbales Gedächtnis" OR "Schreibfähigkeiten" OR "Alphabetisierungsniveau" OR "Autonomie" OR "Empowerment" OR "Unabhängigkeit (Persönlichkeit)" OR "Kontrollüberzeugungen" OR "Selbstbestimmung" OR "Bewusstsein" OR "Aufmerksamkeit" OR “Zielsetzung" OR "Ansprüche" OR "Anspruchsniveau" OR "Bildungsansprüche" OR "Zielorientierung"

	OR "Motivation" OR "Ziele" OR "Intrinsische Motivation" OR "Bedürfnisse" OR "Soziale Motivation" OR "Selbstverwirklichung" OR "Affektive Erziehung" OR "Fähigkeit" OR "Eignungsniveau" OR "Schulbesuch" OR "Schulverweigerung" OR "Schulschwänzen" OR "Schüler- und Studierendenschwund" OR "Schulabgänger" OR "Divergentes Denken" OR "Innovation" OR "Emotionale Entwicklung" OR "Emotionale Intelligenz" OR "Gesundheit" OR "Wohlbefinden" OR "Selbstgesteuertes Lernen" OR "Transfer (Lernen)" OR "Beharrlichkeit" OR "Selbstüberwachung (Persönlichkeit)" OR "Resilienz" OR "Anpassungsfähigkeit (Persönlichkeit)" OR "Bewältigungsverhalten" OR "Emotionale Bewältigung" OR "Emotionale Stabilität" OR "Protektive Faktoren" OR "Psychische Ausdauer" OR "Psychischer Stress")
AND	TX ("Intervention" OR "Group Intervention" OR "School Based Intervention" OR "Curriculum" OR "Program*" OR "Educational Program*" OR "Training" OR "Education" OR "Evaluation" OR "Teaching" OR "Program Evaluation" OR "Course Evaluation" OR "Educational Programs" OR "Educational Program Evaluation" OR "Gruppenintervention" OR "Schulische Intervention" OR "Bildungsprogramme" OR "Training" OR "Evaluation" OR “Kurs” OR "Programmevaluation" OR "Kursevaluation" OR "Evaluation von Bildungsprogrammen")
NOT[a]	SU ("Therapy" OR "Therapie")
Limiters (depending on data base): peer reviewed, academic journal; English or German language; school age or adolescents; empirical, quantitative, longitudinal methods	

Note. [a]The first search of the literature in March 2019 included the Boolean operator NOT. Due to the potential bias when using this operator, the search was rerun without the inclusion of this line as part of the updated search in 2020. The additionally retrieved records were screened according to the criteria outlined.
