# Supplementary material for: Arts in Education: A Systematic Review of Competency Outcomes in Quasi-Experimental and Experimental Studies
Source: Front Psychol. 2021 Apr 15;12:623935. doi: 10.3389/fpsyg.2021.623935 (PMC8082240; doi:10.3389/fpsyg.2021.623935)
Supplement: Supplementary file 2 [file Table_2.pdf]

Supplementary Material

Supplementary Table 2. Study characteristics.

Study	Design[a], control conditions[b]	Sample size	Baseline age range/grade	Program; intensity; duration	Main outcomes and measurement instruments	Power analysis[c]	Attrition[d]
Music							
Brodsky and Sulkin (2011); Study 3	(a) 2<br>(b) 1 (music appreciation guided listening)	n = 51	2nd and 3rd grade	Handclapping song training; 1 x 20 minutes per week; 8 weeks	(1) synchronous hand coordination: Bi-Pat (adapted from Semjen and Vos, 2002)<br>(2) handwriting: Alef-Alef Ktav Yad handwriting quality diagnostic test (Erez and Prush, 1999, as cited in Brodsky and Sulkin, 2011)	2	2
	Results: (1) group x time interaction ANOVA: $F(1, 47) = 23.18$, $p < .001$, $\eta_p^2 = .330$, 95% $CI_{(cal)}$ [.122, .499], with descriptive statistics indicating greater improvements in the experimental group; effect sizes $d_{ppc2(cal)} = 0.89$, and $d_{ppc2(cal)} = 0.68$, for the landscape and portrait formats, respectively. (2) group x time ANOVA: $F(1, 47) = 25.43$, $p < .001$, $\eta_p^2 = .351$, 95% $CI_{(cal)}$ [.139, .516], $d_{ppc2(cal)} = 0.85$, with descriptive statistics indicating greater improvements in the experimental group.						
Bugos and Jacobs (2012)	(a) 2<br>(b) 1 (physical education)	n = 28	6th grade	Composition training; 1 x per week; 4 months	(1) music reading: Music Reading Assessment (MRA, Bugos and Groner, 2009, as	2	NA

Study	Design[a], control conditions[b]	Sample size	Baseline age range/grade	Program; intensity; duration	Main outcomes and measurement instruments	Power analysis[c]	Attrition[d]
					cited in Bugos and Jacobs, 2012)<br>(2) Vocabulary, Arithmetic, Symbol Search, and Digit Coding subtests of the WISC-IV (Wechsler, 2003, as cited in Bugos and Jacobs, 2012)<br>(3) Verbal Fluency subtest of the D-KEFS (Delis et al., 2001, as cited in Bugos and Jacobs, 2012)		
	Results: (1) group x time ANOVA: *n.s.* (2) group x time ANOVA: Arithmetic, $F(1, 26) = 6.64$, $p = .016$, $\eta_p^2{}_{(cal)} = .203$, 95% $CI_{(cal)}$ [.007, .434], $d_{ppc2(cal)} = 0.80$, with descriptive statistics indicating improvements for the experimental and decreases in the control group; Vocabulary, *n.s.*; Digit Coding, $F(1, 26) = 2.80$, $p = .106$, $\eta_p^2{}_{(cal)} = .097$, 95% $CI_{(cal)}$ [0, .325], $d_{ppc2(cal)} = 0.68$, with descriptive statistics indicating greater improvements in the experimental group; Symbol Search, *n.s.* (3) group x time ANOVA: Verbal Fluency, $F(1, 26) = 1.01$, $p = .324$, $\eta_p^2{}_{(cal)} = .037$, 95% $CI_{(cal)}$ [0, .240], $d_{ppc2(cal)} = 0.35$, with descriptive statistics indicating greater improvements in the experimental group.						
Costa-Giomi (1999)	(a) 2<br>(b) 2	$n = 80$	4th grade	Piano lessons; 1 x 30-40 minutes per week; 3 years	(1) self-esteem: Coopersmith Self-Esteem Inventories (1981, as cited in Costa-Giomi, 1999)<br>(2) language and maths skills: Canadian Achievement Test 2 (1992, as cited in Costa-Giomi, 1999)	2	3

Study	Design[a], control conditions[b]	Sample size	Baseline age range/grade	Program; intensity; duration	Main outcomes and measurement instruments	Power analysis[c]	Attrition[d]
					(3) school grades (music, maths, English and French)		
	Results: (1) group x time interaction ANOVA: overall self-esteem, *F*(3, 234) = 2.48, $p < .08$ $\eta_{p(cal)}^2 = .031$, 95% CI [0, .076], due to increases for the music students only; school self-esteem, *F*(3, 234) = 2.83, $p < .05$, $\eta_{p(cal)}^2 = .035$, 95% $CI_{(cal)}$ [0, .082], with *n.s* increases for the music students. (2) group x time interaction ANOVA: math scores, F(2, 158) = 2.45, adj. G-G $p = .09$, $\eta_{p(cal)}^2 = .03$ and when controlling for income, *F*(2, 150) = 3.22, adj. G-G $p < .05$, with *n.s.* higher scores in music students after two years; when including a third group into the analysis (children who did not complete the full three years): total language scores, *F*(4, 156) = 3.31, $p = .01$, $\eta_{p(cal)}^2 = .078$, due to higher post-hoc scores in the music group; language expression, *F*(4, 156) = 2.53, $p < .05$, $\eta_{p(cal)}^2 = .061$ with between-group post-hoc comparisons for language expression *n.s*. (3) group x time interaction ANOVA: music, *F*(3, 198) = 2.89, adj. G-G $p = .04$, $\eta_{p(cal)}^2 = .042$, due to a decline in the control group only; math, *F*(3, 210) = 5.73, adj. G-G $p < .01$, $\eta_{p(cal)}^2 = .076$, post-hoc between-group differences *n.s.*; language subjects, *n.s.*						
Degé et al. (2011)	(a) 2<br>(b) 2	*n* = 34	9-11 years old	Extended music curriculum; 5-7 additional hours per week; 2 years	(1) visual and auditory short-term memory Merk- und Lernfähigkeitstest für 6- bis 16-Jährige (BASIC-MLT; Lepach and Petermann, 2008, as cited in Degé et al., 2011)<br>(2) control measure intelligence: Hamburg-Wechsler-Intelligenztest für Kinder (HAWIKIII; Tewes et al., 2000, as cited in Degé et al., 2011)<br>(3) Musical aptitude: Advanced Measures for Music Audiation	2	3

Study	Design[a], control conditions[b]	Sample size	Baseline age range/grade	Program; intensity; duration	Main outcomes and measurement instruments	Power analysis[c]	Attrition[d]
					(Gordon, 1989, as cited in Degé et al., 2011).		
	Results: (1) group x time interaction ANCOVA (covariate: extracurricular schooling): visual memory, $F(1, 31) = 4.20$, $p = .049$, $\eta_p^2 = .12$, due to significant improvements after one year for the music group only; auditory memory, $F(1, 31) = 9.02$, $p = .005$, $\eta_{p\,(cal)}^2 = .23$, due to significant improvements in the music group only. (2) group x time interaction ANOVA: *n.s.* (3) group x time interaction ANOVA: *n.s.*						
Guo et al. (2018)	(a) 2<br>(b) 2	$n = 40$	6-8 years old	Keyboard harmonica lessons; 2 x 25 minutes per week; 6 weeks	(1) vocabulary, processing speed, and working memory: Japanses version of the Wechsler Intelligence Scale for Children (Ueno et al., 2010, as cited in Guo et al., 2018)<br>(2) verbal ability: rapid automized naming test (RAN; Norton and Wolf, 2012)<br>(3) inhibitory control: Go/No-go task (Moreno et al., 2011)	2	1
	Results: (1) group x time interaction ANOVA: vocabulary, $F(1, 38) = 1.33$, $p = .248$, $\eta^2 = .034$, 95% $CI_{(cal)}$ [0, .194], $d_{ppc2} = -0.33$, with descriptive statistics indicating a greater improvement in the control group; processing speed, *n.s.*, Letter-Number Sequencing subtest, *n.s.*; working memory, Digit Span subtest, $F(1, 38) = 4.53$, $p = .034$, $\eta^2 = .107$, 95% $CI_{(cal)}$ [0, .299], $d_{ppc2} = 0.64$, with descriptive statistics indicating a greater improvement in the experimental group; Digit Span Backwards, $F(1, 38) = 6.35$, $p = .015$, $\eta^2 = .143$, 95% $CI_{(cal)}$ [.004, .340], $d_{ppc2} = 0.87$, with descriptive statistics indicating increases in the experimental and decreases in the control group; Digit Span Forwards, $F(1, 38) = 0.46$, $p = .491$, $\eta^2 = .012$, 95% $CI_{(cal)}$ [0, .145]. (2) group x time interaction ANOVA: $F(1, 38) = 1.56$, $p = .212$, $\eta^2 = .039$, 95% $CI_{(cal)}$ [0, .206], $d_{ppc2} = 0.23$. (3) group x time interaction ANOVA: *n.s.*						

Study	Design[a], control conditions[b]	Sample size	Baseline age range/grade	Program; intensity; duration	Main outcomes and measurement instruments	Power analysis[c]	Attrition[d]
Rabinowitch et al. (2013)	(a) 1<br>(b) 1 (interactive game session or no treatment)	*n* = 52	8-11 years old	Musical group interaction; 1 x 1 hour per week; 3-9 months	(1) empathy (self-report): Index of Empathy (Bryant, 1982)<br>(2) empathy (non-verbal test): Matched Faces (based on de Wied et al., 2005)<br>(3) verbal abilities: Similarity and Vocabulary subtests of the Wechsler Intelligence Scale for Children (Wechsler, 1974, as cited in Rabinowitch et al., 2013)	2	NA
	Results: (1) group x time interaction ANOVA: $F(1, 50) = 3.90$, $p = .054$, $\eta_{p}^{2}{}_{(cal)} = .072$, 95% $CI_{(cal)}$ [0, .231], due to significant improvements for the experimental group only, $t(22) = 3.51$, $p < .01$, $d_{(cal)} = 0.73$. (2) group x time interaction ANOVA: *n.s.* (3) group x time interaction ANOVA: *n.s.*						
Rickard et al. (2012); Study 2	(a) 1<br>(b) 1 (drama training or no treatment)	*n* = 84	Primary school (age: $M = 10.92$ years, $SD = 0.58$)	Music training; 1 x 1 extra hour per week; one semester	(1) Rosenberg self-esteem scale (Rosenberg, 1965, as cited in Rickard et al., 2012)<br>(2) Social Skills Rating System (Student and Parent Form; Gresham and Elliot, 1990, as cited in Rickard et al., 2012),<br>(3) Aggression Questionnaire (Buss and Warren, 2000, as cited in Rickard et al., 2012),	2	3

Study	Design[a], control conditions[b]	Sample size	Baseline age range/grade	Program; intensity; duration	Main outcomes and measurement instruments	Power analysis[c]	Attrition[d]
					(4) Reynolds Child Depression Scale (Reynolds, 1989, as cited in Rickard et al., 2012). (5) Attitudes to School Survey (Department of Education and Early Childhood Development, 2006, as cited in Rickard et al., 2012). (6) academic measures from the school		
	Results: (1) group x time interaction ANOVA: *n.s.* (2) group x time interactions ANOVA: *n.s.* (3) group x time interaction ANOVA: $F(2, 77) = 2.46$, $p = .092$, $\eta_p^2{}_{(cal)} = .060$, 95% $CI_{(cal)}$ [0, .169]; trends in descriptive data indicate an increase for music and drama students whilst control group student decreased. (4) group x time interaction ANOVA: *n.s.* (5) group x time interaction ANOVA: *n.s.* (6) group x time interaction ANOVA: reading, $F(2,77) = 3.39$, $p = .039$, $\eta_p^2 = .081$, 95% $CI_{(cal)}$ [0, .199]; trends for greater improvement in the control group, but post-hoc tests *n.s.*; working mathematically, $F(2,77) = 3.53$, $p = .034$, $\eta_p^2 = .084$, 95% $CI_{(cal)}$ [0, .203]; trends for greater improvement in the music and drama groups compared to the control group, but post-hoc tests *n.s.*						
Rickard et al. (2010)	(a) 2<br>(b) 2	$n = 142$	Primary school (mean age 8.69 years)	Intensive string music training programme; 1 x 1 hour per week; 2 years	(1) verbal learning, immediate memory, delayed memory, verbal recognition, and digit span (for attention): The Children's Memory Scale (Cohen, 1997, as cited in Rickard et al., 2010)	2	3

Study	Design[a], control conditions[b]	Sample size	Baseline age range/grade	Program; intensity; duration	Main outcomes and measurement instruments	Power analysis[c]	Attrition[d]
					(2) subtests Immediate Visual Memory and Visual Performance of the Benton's Visual Retention Test (Sivan, 1992, as cited in Rickard et al., 2010)<br>(3) music tests for music group only (no test referenced)		
	Results: (1) group x time interaction ANOVA: verbal learning, $F(2, 208) = 8.18$, $p < .001$, $\eta_p^2 = .073$, 95% $CI_{(cal)}$ [.016, .143] across three measurement points; greater improvement in the music group after the first year, $F(1, 133) = 14.98$, $p < .001$, $\eta_p^2 = .101$, 95% $CI_{(cal)}$ [.025, .204]; immediate verbal recall, $F(2, 210) = 3.82$, $p = .024$, $\eta_p^2 = .035$, 95% $CI_{(cal)}$ [<.001, .090] across three measurements points; greater improvements for the music group after the first year, $F(1, 133) = 4.75$, $p = .031$, $\eta_p^2 = .034$, 95% $CI_{(cal)}$ [0, .113], greater improvement for control group between first and second year, $F(1, 105) = 4.68$, $p = .033$, $\eta_p^2 = .043$, 95% $CI_{(cal)}$ [0, .138]; digit span, greater improvement from first to second year for the music group, $F(1, 100) = 4.30$, $p = .041$, $\eta_p^2 = .041$, 95% $CI_{(cal)}$ [0, .138]. (2) group x time interaction ANOVA: immediate visual memory, *n.s.*; visual performance, $F(2, 212) = 3.16$, $p = .045$, $\eta_p^2 = .029$, 95% $CI_{(cal)}$ [0, .080] across three measurement points; decline in the control group from between the first and second year, $F(1, 106) = 6.60$, $p = .012$, $\eta_p^2 = .059$, 95% $CI_{(cal)}$ [.003, .161]. (3) dependent t-test: $t(62) = 7.20$, $p < .05$, $d_{(cal)} = 0.91$, due to improvements from baseline.						
Roden et al. (2014a)	(a) 2<br>(b) 1 (extended science curriculum)	$n = 50$	7-8 years old	Music instrumental training; 1 x 45 minutes per week; 18 months	Test batteries by Hasselhorn et al. (2012, as cited in Roden, Grube et al., 2014) assessing working memory components:<br>(1) visualspatial sketchpad (subtest Corsi Block and Matrix Span)	1	NA

Study	Design[a], control conditions[b]	Sample size	Baseline age range/grade	Program; intensity; duration	Main outcomes and measurement instruments	Power analysis[c]	Attrition[d]
					(2) phonological loop (subtests One-Syllable Word Span and Nonword Recall)<br>(3) central executive (subtests Counting Span, Complex Span and Color Span Backwards)		
	Results: (1) group x time interaction ANCOVA (covariates age and intelligence): *n.s.* (2) group x time interaction ANCOVA (covariates age and intelligence): One-Syllable Word Span, $F(1.81, 83.33) = 12.68$, adj. H-F $p < .001$, $\eta_p^2 = .22$, across three measurement points; music group improved for both time intervals after the first and second year, $t(24) = 4.23$, $p < .001$, $d = 0.85$, and $t(24) = 5.37$, $p < .001$, $d = 1.11$; Nonword Recall, $F(2, 92) = 3.42$, $p < .05$, $\eta_p^2 = .07$, across three measurement points; music group improved in both time intervals, $t(24) = 3.62$, $p = .001$, $d = 0.73$, and $t(24) = 2.15$, $p < .05$, $d = 0.43$; the control group only improved in the first year, $t(24) = 2.28$, $p < .05$, $d = 0.46$. (3) group x time interaction ANCOVA (covariates age and intelligence): Counting Span, $F(2, 92) = 4.91$, $p < .05$, $\eta_p^2 = .10$; both groups improved in the first year, $t(24) = 3.35$, $p = .003$, $d = 0.68$, and $t(24) = 2.65$, $p = .01$, $d = 0.53$; across both years, only the improvement in the music group was significant, $t(24) = 6.63$, $p < .001$, $d = 1.33$; Complex Span, $F(1.73, 79.33) = 6.35$, adj. H-F $p < .05$, $\eta_p^2 = .12$; music group improved in both time intervals, $t(24) = 4.05$, $p < .001$, $d = 0.82$, and $t(24) = 2.79$, $p = .010$, $d = .58$; Color Span Backwards, *n.s.* When controlling for phonological loop scores, the interaction effects on the central executive became *n.s.*, indicating that performance on the central executive was mediated by working memory on the phonological loop.						
Roden et al. (2014b)	(a) 2<br>(b) 1 (extended science curriculum)	$n = 345$	7-8 years old	Music instrumental training; 1 x 45 minutes per week; 18 months	(1) music learning: Immediate Measures of Music Audiation instrument (Gordon, 1979, as cited in Roden, Könen et al., 2014)	2	NA

Study	Design[a], control conditions[b]	Sample size	Baseline age range/grade	Program; intensity; duration	Main outcomes and measurement instruments	Power analysis[c]	Attrition[d]
					(2) visual attention: d2 Test of Attention (Brickenkamp, 2002, as cited in Roden, Könen, et al., 2014) (3) information processing speed: Zahlen-Verbindungs-Test (Oswald and Roth, 1978, as cited in Roden, Könen et al., 2014).		
	Results: Structural equation models: model fit confirmed acceptable on descriptive data, $\chi2$ (96) = 249.04, CFI = .965 for visual attention, and $\chi2$ (120) = 131.17, CFI = .997 for processing speed. (1) Rhythm: significant higher improvements for music group in the first year, $\Delta M_{control} - \Delta M_{music} = - 4.29$, $SE = 1.77$, $p = .015$, $d = 0.29$, *n.s.* for the second year; tonal (not measured at baseline): *n.s.* for the second year. (2) Visual attention: significant higher improvements in the control group in the first and second year, $\Delta M_{control} - \Delta M_{music} = 0.46$, $SE = 0.22$, $p = .037$, $d = 0.24$, and $\Delta M_{control} - \Delta M_{music} = .98$, $SE = 0.21$, $p < .001$, $d = 0.56$. (3) Processing speed: significant higher improvements for the music group in the second year, $\Delta M_{control} - \Delta M_{music} = - 0.05$, $SE = 0.02$, $p = .030$, $d = 0.30$.						
Roden et al. (2012)	(a) 2 (b) 1 (extended science curriculum)	$n = 73$	7-8 years old	Music instrumental training; 1 x 45 minutes per week; 18 months	(1) verbal memory: Verbal Learning, Verbal Delayed Recall, and Verbal Recognition (Verbaler Merk- und Lernfähigkeitstest; Helmstaedter et al., 2001, as cited in Roden et al., 2012) (2) visual memory (subtest Corsi Block and Matrix Span)	1	NA

Study	Design[a], control conditions[b]	Sample size	Baseline age range/grade	Program; intensity; duration	Main outcomes and measurement instruments	Power analysis[c]	Attrition[d]
	Results: (1) group x time interactions ANCOVA (covariates age and intelligence): Verbal Learning, $F(3.63, 123.4) = 5.19$, adj. G-G $p = .001$, $\eta_p{}^2{}_{(cal)} = .132$, significant improvement for music group on Verbal Learning scores in the first and second year, $t(24) = 5.71$, $p < .001$, $d = 1.16$, and, $t(24) = 6.43$, $p < .001$, $d = 1.35$. Changes *n.s.* in science group; untreated control group improved in both time intervals, $t(22) = 5.14$, $p < .001$, $d = 1.07$, and $t(22) = 4.22$, $p < .001$, $d = 0.88$; Verbal Delayed Recall, $F(4, 136) = 3.17$, $p = .016$, $\eta_p{}^2{}_{(cal)} = .085$; the music group improved in both time intervals, $t(24) = 5.66$, $p < .001$, $d = 1.27$, and $t(24) = 4.92$, $p < .001$, $d = 1.06$; the science group only significantly improved after the first year, $t(24) = 2.46$, $p = .021$, $d = 0.49$ and the untreated control group only for the last interval, $t(22) = 3.33$, $p < .05$, $d = 0.70$; Verbal Recognition, $F(3.05, 103.56) = 2.72$, adj. G-G $p = .048$, $\eta_p{}^2{}_{(cal)} = .074$; the music group improved after the first year, $t(24) = 3.09$, $p = .005$, $d = 0.67$, and during the last six months, $t(24) = 3.01$, $p = .006$, $d = 0.65$. No significant improvements for the other groups. (2) group x time interaction ANCOVA: *n.s.*						
Roden et al. (2016)	(a) 2<br>(b) 1 (extended science curriculum)	$n = 34$	7-8 years old	Music instrumental training; 1 x 45 minutes per week; 18 months	(1) scores on computer version of Point-Subtraction Aggression Game (Pelham et al., 1991)<br>(2) affective stress response: German version of the Positive and Negative Affect Schedule (Krohne et al., 1996)<br>(3) physiological stress measures: systolic and diastolic blood pressure, heart rate, and cortisol concentrations in the saliva	2	NA

Study	Design[a], control conditions[b]	Sample size	Baseline age range/grade	Program; intensity; duration	Main outcomes and measurement instruments	Power analysis[c]	Attrition[d]
	Results: (1) group x time interaction ANOVA: $F(1, 29) = 5.69$, $p = .024$, $\eta_p^2{}_{(cal)} = .164$, 95% $CI_{(cal)}$ [.001, .387], $d_{ppc2} = - 0.94$; control group increased in their aggression scores, $t(16) = 2.54$, $p_{(cal)} < .05$, $d = 1.27$, music group changes *n.s.* (2) group x time interaction ANOVA: *n.s.* (3) group x time interaction ANOVA: *n.s.*						
Schellenberg et al. (2015)	(a) 2<br>(b) 2	$n = 84$	3rd and 4th grade	Ukulele instruction; 1 x 40 minutes per week; 10 months	(1) Test of Emotion Comprehension (Pons and Harris, 2000, as cited in Schellenberg et al., 2015)<br>(2) Child-Report Sympathy Scale (Zhou et al., 2003, as cited in Schellenberg et al., 2015)<br>(3) prosocial behaviour: Social Behavior Questionnaire (Tremblay et al., 1991)<br>(4) vocabulary: Peabody Picture Vocabulary Test (Dunn and Dunn, 2006, as cited in Schellenber et al., 2015)	2	NA
	Results: (1) group x time (x initial performance) interactions ANOVA: *n.s.* (2) time x group x initial performance interaction ANOVA: $F(1, 80) = 4.94$, $p = .029$, $\eta_p^2 = .058$, 95% $CI_{(cal)}$ [0, .177]; time x group interaction for low scorers, $F(1, 41) = 5.07$, $p = .030$, $\eta_p^2 = .108$, 95% $CI_{(cal)}$ [0, .297], with significant improvements for music students, $t(17) = 3.45$, $p = .003$, $d_{(cal)} = 0.81$; for high scorers *n.s.* (3) time x group x initial performance interaction ANOVA: $F(1, 80) = 10.45$, $p = .002$, $\eta_p^2 = .116$, 95% $CI_{(cal)}$ [.017, .251]; time x group interaction for low scorers, $F(1, 42) = 9.23$, $p = .004$, $\eta_p^2 = .180$, 95% $CI_{(cal)}$ [.020, .370], with significant improvements for music students, $t(13) = 3.49$, $p = .008$, $d_{(cal)} = 0.93$. (4) group x time (x initial performance) interactions ANOVA: *n.s.*						

Study	Design[a], control conditions[b]	Sample size	Baseline age range/grade	Program; intensity; duration	Main outcomes and measurement instruments	Power analysis[c]	Attrition[d]
Drama							
Köksal Akyol (2018)	(a) 1<br>(b) 2	*n* = 46	1st grade	Drama classes; 1 x 2 hours per week; 12 weeks	Multiple intelligences with the subtests of the Turkish version of the Teele Inventory for Multiple Intelligences (Göğebakan, 2003, as cited in Köksal Akyol, 2018)	2	NA
	Results: group x time interaction ANOVA: Verbal-Linguistic Intelligence, *n.s.*; Mathematical-Logical, *n.s.*; Visual-Spatial, *n.s.*; Musical-Rhythmic Intelligence, *n.s.*; Bodily Kinaesthetic, *n.s.*; Intrapersonal Intelligence, *n.s.*; Interpersonal Intelligence, $F(1, 44) = 0.65$, $p = .423$, $\eta_{p}^{2}{}_{(cal)} = .015$, 95% $CI_{(cal)}$ [0, .143], $d_{ppc2} = - 0.28$, with descriptive statistics indicating decreases in the experimental and increases in the control group.						
Sowden et al. (2015); Study 2	(a) 1<br>(b) 1 (non-improvisation activities)	*n* = 34	10-11 years old	Drama improvisation; one session only; no duration reported	Incomplete Figures Tasks of the Torrance Tests of Creative Thinking (Torrance, 1974, as cited in Sowden, Clements et al., 2015) with subscores originality, elaboration, and abstractness	2	NA
	Results: ANCOVA on post-test scores (covariate elaboration baseline scores): originality, $F(1, 31) = 7.09$, $p = .012$, $\eta_p^2 = .19$, with higher post-test scores for improvisation students; elaboration, $F(1, 31) = 6.24$, $p = .018$, $\eta_p^2 = .17$, with higher post-test scores for improvisation students; abstractness: *n.s.*						
Walsh-Bowers (1992)	(a) 2<br>(b) 2	*n* = 104	6th grade	Drama program; 1 x 40	(1) Peer Interaction Scale (Wheeler and Ladd, 1982), subdimensions cooperation and	2	NA

Study	Design[a], control conditions[b]	Sample size	Baseline age range/grade	Program; intensity; duration	Main outcomes and measurement instruments	Power analysis[c]	Attrition[d]
				minutes per week; 14 weeks	conflict, (2) School Pressure Scale (adapted version, Elias et al., 1986)<br>(3) Teacher-Child Rating Scale (Hightower et al., 1986); subscales problems and competencies<br>(4) Parent Rating of Social Skills; subscales problems and strengths		
	Results: (1) main effect of group on change scores: *n.s.*; (2) main effect of group on change scores: *n.s.*; (3) main effect of group on change scores: problems, $F(1, 100) = 29.66$, $p < .001$, $\eta_{p(cal)}^2 = .229$, 95% $CI_{(cal)}$ [.098, .358], effect based on pre-post-test scores $d_{ppc2} = -1.58$, due to a stronger increase in the control group; competencies, $F(1, 100) = 14.16$, $p < .001$, $\eta_{p(cal)}^2 = .124$, 95% $CI_{(cal)}$ [.028, .247], effect based on pre-post-test scores $d_{ppc2} = 0.78$, due to a stronger decrease in the control group. (4) main effect of group on change scores: problems, *n.s.*, effect based on pre-post-test scores $d_{ppc2} = -0.34$; strengths, $F(1, 44) = 11.45$, $p = .002$, $\eta_{p(cal)}^2 = .206$, 95% $CI_{(cal)}$ [.035, .392], effect based on pre-post-test scores $d_{ppc2} = 0.68$, due to an increase in the drama group.						
Walsh-Bowers and Basso (1999); Study 1	(a) 2<br>(b) 2	$n = 44$	7th grade	Drama program; 5 x 1 hour per week; 15 weeks	(1) Peer Interaction Scale (Wheeler and Ladd, 1982), subdimensions cooperation and conflict<br>(2) teacher report of peer problems: Peer Evaluation Inventory (Pekarik et al., 1976)	2	NA

Study	Design[a], control conditions[b]	Sample size	Baseline age range/grade	Program; intensity; duration	Main outcomes and measurement instruments	Power analysis[c]	Attrition[d]
					(3) Parent Rating of Social Skills; subscales problems and strengths		
	Results: (1) main effect of group on change scores: conflict, *n.s.*; cooperation, *n.s.*, effect based on pre-post-test scores d_{ppc2} = 0.22 (2) measure excluded due to confounding effects in control group; (3) main effect of group on change scores: problems, $F(1, 43) = 8.17$, $p = .007$, $\eta_p^2{}_{(cal)} = .160$, 95% $CI_{(cal)}$ [.014, .347], effect based on pre-post-test scores d_{ppc2} = - 0.68, due to a decrease in the drama compared to an increase in the control group; strengths, $F(1, 43) = 3.72$, $p = .061$, $\eta_p^2{}_{(cal)} = .080$, 95% $CI_{(cal)}$ [0, .254], effect based on pre-post-test scores d_{ppc2} = 0.59, due to an increase in the drama compared to a decrease in the control group.						
Walsh-Bowers and Basso (1999); Study 2	(a) 2<br>(b) 2	$n = 75$	7th grade	Drama program; 5 x 1 hour per week; 15 weeks	(1) Peer Interaction Scale (Wheeler and Ladd, 1982), subdimensions cooperation and conflict<br>(2) teacher report of peer problems: Peer Evaluation Inventory (Pekarik et al., 1976), subscales problems and strengths<br>(3) Parent Rating of Social Skills; subscales problems and strengths	2	NA
	Results[e]: (1) main effect of group on change scores: *n.s.*; (2) main effect of group on change scores: problems, $F(3, 71) = 2.27$, $p < .05$, effect based on pre-post-test scores d_{ppc2} = - 0.39, due to increases in the drama group only; strengths: $F(3, 71) = 23.95$, $p < .001$, effect based on pre-post-test						

Study	Design[a], control conditions[b]	Sample size	Baseline age range/grade	Program; intensity; duration	Main outcomes and measurement instruments	Power analysis[c]	Attrition[d]
	scores d_{ppc2} = - 0.89, due to a decrease in the music compared to improvements in the control group. (3) measure excluded due to a low return rate of forms.						
Dance							
Bollimbala et al. (2019)	(a) 1<br>(b) 2	*n* = 34	6th and 7th grade	Folk dance physical activity; one 20-minute session only	(1) convergent thinking: Remote Association Test (Mednick, 1962)<br>(2) divergent thinking: Guildford Alternate Uses (Guilford, 1967)	1	NA
	Results[f]: (1) main effect on post-test scores, full sample: $F(1, 32) = 4.46$, $p = .04$, $\eta_p^2 = .12$, 95% $CI_{(cal)}$ [0, .33], due to higher post-test scores in the intervention group, effect based on pre-post-test scores d_{ppc2} = 0.35; low BMI sample: *n.s.*, effect based on pre-post-test scores d_{ppc2} = 0.15; normal BMI sample: $F(1, 15) = 6.48$, $p = .02$, $\eta_p^2 = .30$, 95% $CI_{(cal)}$ [0; 0.56], due to higher post-test scores in the intervention group; effect based on pre-post-test scores d_{ppc2} = 0.41-0.49; (2) main effect on post-test scores, fluency, full sample: *n.s.*; low BMI sample: *n.s.*, effect based on pre-post-test scores d_{ppc2} = - 0.38, with descriptive statistics indicating decreases in the treatment and increases in the control group; normal BMI sample: $F(1, 15) = 6.39$, $p = .02$, $\eta_p^2 = .29$, 95% $CI_{(cal)}$ [0;.56], due to higher post-test scores in the intervention group; effect based on pre-post-test scores d_{ppc2} = 0.41-0.43; flexibility, full sample: $F(1, 32) = 3.62$, $p = .06$, $\eta_p^2 = .10$, 95% $CI_{(cal)}$ [0, .30], effect based on pre-post-test scores d_{ppc2} = -0.37, with descriptive statistics indicating decreases in the treatment and increases in the control group; low BMI sample: *n.s.*, effect based on pre-post-test scores d_{ppc2} = - 0.68, with descriptive statistics indicating decreases in the treatment and increases in the control group; normal BMI sample: $F(1, 15) = 23.54$, $p = .001$, $\eta_p^2 = .61$, 95% $CI_{(cal)}$ [.22; .76], due to higher post-test scores in the intervention group; effect based on pre-post-test scores d_{ppc2} = 0.35-0.36; originality, full sample: *n.s.*; low BMI sample: *n.s.*; normal BMI sample: *n.s.*, $\eta_p^2 = .16$, effect based on pre-post-test scores d_{ppc2} = 0.40-0.43, with descriptive statistics indicating improvements in the intervention group only.						
Kreutzmann et al. (2018)	(a) 2<br>(b) 2	*n* = 606	5th to 9th grade	Dance intervention; 1	(1) intrapsychic measure of social belonging: The Inclusion	2	NA

Study	Design[a], control conditions[b]	Sample size	Baseline age range/grade	Program; intensity; duration	Main outcomes and measurement instruments	Power analysis[c]	Attrition[d]
				x 90 minutes per week; one or two semesters	of Other in the Self scale (Aron et al., 1992)<br>(2) extrapsychic measure of social belonging/ social acceptance: indegree and outdegree centrality		
	Results: multilevel longitudinal mediation analyses: (1) direct effect, *z* = 1.94, *p* = .053, *d* = 0.16; partial mediation via outdegree centrality, indirect effect, *z* = 2.86, *p* = .004, *d* = 0.14, direct effect, *z* = 2.02, *p* = .044, *d* = 0.09; partial mediation via indegree centrality, indirect effect, *z* = 2.19, *p* = .029, *d* = 0.19, direct effect, *z* = 2.17, *p* = .030, *d* = 0.18. However, using Helmert contrast coding, the only mediating effect could be found via outdegree centrality, *z* = 1.99, *p* = .047, *d* = 0.09; (2) direct effect outdegree, *z* = 3.12, *p* = .002, *d* = 0.26, and indegree centrality, *z* = 2.41, *p* = .016, *d* = 0.20.						
Zander et al. (2014)	(a) 2<br>(b) 2	*n* = 361	5th to 9th grade	Dance intervention; 1 x 90 minutes per week; one or two semesters	(1) affective and collaborative degree centrality: proportions of reciprocal nominations to the total number of possible nominations<br>(2) opposite sex nominations: proportions to the total number opposite sex nominations possible	2	2
	Results: multiple regression analysis: (1) affective degree centrality (controlling for music grades and previous dance experience): *n.s.*; collaborative degree centrality: significant treatment x gender interaction effect, *B* = - 0.08, *SE* = 0.03, *t*(353) = - 3.22, *p* = .001, *d* = - 0.34, due to a significant increase in collaborative relationships for boys in the intervention group to the same level as girls whilst boys in the control group						

Study	Design[a], control conditions[b]	Sample size	Baseline age range/grade	Program; intensity; duration	Main outcomes and measurement instruments	Power analysis[c]	Attrition[d]
	stayed at a lower level. (2) comparing post-test scores of boys in the intervention and control groups: ingoing nominations, $t(169) = 1.68$, $p = .10$, $d = 0.26$, outgoing nominations $t(169) = 1.97$, $p = .05$, $d = 0.30$, opposite-sex nominations, $t(19) = 2.59$, $p = .02$, $d = 1.19$, due to the boys in the intervention group scoring higher at post-tests on the three measures.						
Visual arts							
Catterall and Peppler (2007)	(a) 2<br>(b) 2	$n = 179$	3rd grade	Visual arts classes; one group: 2 x 90 minutes per week; 20 weeks; other group: 1 x 1 hour per week; 30 weeks	(1) General self-concept, general self-efficacy beliefs, internal and external success attribution and worldviews with scales developed by authors<br>(2) creativity dimensions originality, fluency, flexibility and elaboration; self-report based on the Torrance test of creativity (Abedi, 2002; Auzmendi et al., 1996)	2	2
	Results: (1) Self-efficacy: percentages of significant improvements greater in visual arts group, $\chi 2$ (0, .01) > 6.635; $p < .01$, effect based on pre-post-test scores $d_{ppc2} = 0.54$; $\chi 2$-tests: general self-concept, *n.s.*; success attributions, *n.s.*; worldviews, *n.s.* (2) Originality: percentages of significant improvements greater in the visual arts group, effect based on pre-post-test scores $d_{ppc2} = 0.81$; $\chi 2$-tests: elaboration, *n.s.*; flexibility, *n.s.*; fluency, *n.s.*						
Comparisons between different art domains							

Study	Design[a], control conditions[b]	Sample size	Baseline age range/grade	Program; intensity; duration	Main outcomes and measurement instruments	Power analysis[c]	Attrition[d]
François et al. (2013)	(a) 1<br>(b) 1 (painting)	*n* = 24	8 years old	Musical training; 1-2 x 45 minutes per week, 2 years	(1) performance on artificial language test (percentage of correct responses)<br>(2) sensitivity in speech segmentation: familiarity effect as measured by the difference between ERPs for familiar and unfamiliar items	2	3
	Results: (1) group x time interaction 2 x 3 ANOVA: $F(2, 44) = 3.4$, $p = .04$, $\eta_p{}^2{}_{(cal)} = .134$, 95% $CI_{(cal)}$ [0, .300]; performance in experimental group significantly better than chance at both follow-ups, however no significant difference between the two follow-ups; control group did not perform better than chance at any point. (2) group main effect on post-test scores: familiarity effect was significantly higher for the music group compared to the painting group, $F(1, 20) = 7.9$, $p = .01$, $\eta_p{}^2{}_{(cal)} = .283$, 95% $CI_{(cal)}$ [.017, .524].						
Freeman et al. (2003)	(a) 1<br>(b) 1 (music classes)	*n* = 185	3rd and 4th grade	Creative drama; 1 x 40 minutes per week; 18 weeks	(1) self-rating of self-image, academic self-concept and social self-concept on the Student Self-concept Scale (Gresham et al., 1993, as cited in Freeman et al., 2003).<br>(2) teacher rating of social skills and problem behaviour on the Social Skills Rating System (Gresham and Elliot,	2	3

Study	Design[a], control conditions[b]	Sample size	Baseline age range/grade	Program; intensity; duration	Main outcomes and measurement instruments	Power analysis[c]	Attrition[d]
					1990, as cited in Freeman et al., 2003)		
	Results: (1) ANCOVA main treatment effect on post-test scores (controlling for pretest scores): *n.s.*; self-image, effect based on pre-post-test scores d_{ppc2} = 0.26, with descriptive statistics indicating greater improvements for drama students; academic self-concept, effect based on pre-post-test scores d_{ppc2} = 0.32, with descriptive statistics indicating greater improvements for drama students; social self-concept, effect based on pre-post-test scores d_{ppc2} = - 0.21, with descriptive statistics indicating greater improvements for music students; (2) ANCOVA main treatment effect on post-test scores (controlling for pretest scores): *n.s.*						
Hogenes et al. (2016)	(a) 2<br>(b) 1 (music reproduction)	n = 133	5th and 6th grade	Music composition training; 1 x 45 minutes per week; six months	(1) school tests in language, reading and mathematics<br>(2) intelligence: Raven Standard Progressive Matrices (Raven, 2004, as cited in Hogenes et al., 2016)<br>(3) singing abilities: Musical Abilities Test for Singing and Listening (developed by the authors)	2	2
	Results: MANCOVA followed by ANCOVAs (covariates age and pre-test results): (1) *n.s.*, except reading comprehension: composition group had greater covariate adjusted post-test scores, $F(1, 112) = 7.42$, $p = .007$, $\eta_p^2 = .06$. (2) *n.s.* (3) *n.s.*						
Rickard et al. (2012); Study 1	(a) 2<br>(b) 1 (increased drama or arts lessons)	n = 111	10-13 years old	Increased music lessons; NA; six months	(1) verbal memory: Word Pairs Subtest of the Children's Memory Scale (Cohen, 1997, as cited in Rickard et al., 2012)	2	2

Study	Design[a], control conditions[b]	Sample size	Baseline age range/grade	Program; intensity; duration	Main outcomes and measurement instruments	Power analysis[c]	Attrition[d]
					(2) intelligence: Verbal and Non-verbal Intelligence Scales of the Kaufman Brief Intelligence Test (Kaufman and Kaufman, 2004, as cited in Rickard et al., 2012)<br>(3) Culture-Free Self-Esteem Inventory (Battle, 2002, as cited in Rickard et al., 2012)<br>(4) attitudes to school: School Life Questionnaire (Australian Council for Educational Research, 1984, as cited in Rickard et al., 2012)<br>(5) engagement within the arts classes: Motivation and Engagement Scale (Martin, 2008, as cited in Rickard et al., 2012)		
	Results: (1) group x time interaction ANOVA: *n.s.*., immediate recall verbal memory, *n.s.*, d_{ppc2} = 0.31, with descriptive statistics indicating greater improvement in music compared to drama students; (2) group x time interaction ANOVA: non-verbal intelligence, $F(1, 76) = 3.87$, $p = .053$, $\eta_p^2 = .05$, 95% $CI_{(cal)}$ [0, .166], d_{ppc2} = 1.56, with descriptive statistics indicating greater improvement in music compared to drama students; verbal intelligence, *n.s.*; (3) group x time interaction ANOVA: *n.s.;* academic self-esteem, *n.s*, d_{ppc2} = - 0.22, with descriptive statistic indicating greater decreases in music students compared to drama students; (4) group x time interactions ANOVA: *n.s.*; total attitude score, *n.s.*, d_{ppc2} = - 0.36, with						

Study	Design[a], control conditions[b]	Sample size	Baseline age range/grade	Program; intensity; duration	Main outcomes and measurement instruments	Power analysis[c]	Attrition[d]
	descriptive statistics indicating greater improvements for drama compared to music students; attitudes towards social integration, *n.s.*, d_{ppc2} = - 0.22, with descriptive statistics indicating greater improvements for drama compared to music students; attitudes towards teaching, d_{ppc2} = - 0.51, with descriptive statistics indicating greater improvements for drama compared to music students; status among peers d_{ppc2} = - 0.60, *n.s.*, with descriptive statistics indicating greater improvements for drama compared to music students; (5) group x time interaction ANOVA: adaptive thoughts, $F(2, 81) = 15.24$, $p < .001$, $\eta_p^2 = .273$, 95% $CI_{(cal)}$ [.112, .405], d_{ppc2} = - 1.55, due to a drop in music students not observed in drama students,; adaptive behaviours, $F(2, 81) = 6.67$, $p = .002$, $\eta_p^2 = .141$, 95% $CI_{(cal)}$ [.022, .270], d_{ppc2} = - 1.05, due to a drop in music students not observed in drama students; maladaptive thoughts, *n.s.*, d_{ppc2} = 0.53, with descriptive statistics indicating a decrease for drama students only; maladaptive behaviour, $F(2, 81) = 7.06$, $p = .001$, $\eta_{p(cal)}^2 = .148$, 95% $CI_{(cal)}$ [.026, .278], d_{ppc2} = 1.15, with descriptive statistic indicating increases for the music and decreases for the drama students.						

Note. Report of non-significant and trivial effect sizes omitted to *n.s.* (not significant). Index *(cal)* indicates additional effect size calculations based on the data in the original article (Lenhard and Lenhard, 2016; Stangroom, 2019; Uanhoro, 2017). d_{ppc2} = Effect size sensu Morris (2008). [a]Design: 1 = experimental pretest-posttest-design; 2 = quasi-experimental pretest-posttest-design. [b]Control conditions: 1 = Treated control group; 2 = Untreated control group. [c]Power analysis: A priori power analysis reported? 1 = yes; 2 = no. [d]Attrition (according to Schulz & Grimes, 2002): 1 = low attrition bias (< 5%); 2 = medium attrition bias (5-20%); 3 = serious attrition bias (> 20%). [e]Additional effect size calculations not attempted here due to inconsistencies in the degrees of freedom. [f]Due to a lack of information on subsample sizes, a range of effects sizes is given based on calculations with different subsample sizes.

References

Abedi, J. (2002). A latent-variable modeling approach to assessing reliability and validity of a creativity instrument. *Creat. Res. J.* 14, 267–276. doi: 10.1207/S15326934CRJ1402_12

Aron, A., Aron, E. N., and Smollan, D. (1992). Inclusion of Other in the Self scale and the structure of interpersonal closeness. *J. Personl. Soc. Psychol.* 63, 596–612. doi: 10.1037/0022-3514.63.4.596AQ

Auzmendi, E., Villa, A., and Abedi, J. (1996). Reliability and validity of a newly constructed multiple choice creativity instrument. *Creat. Res. J.* 9, 89–95. doi: 10.1207/s15326934crj0901_8

Bollimbala, A., James, P. S., and Ganguli, S. (2019). Impact of acute physical activity on children's divergent and convergent thinking: the mediating role of a low body mass index. *Percept. Mot. Skills.* 126, 603–622. doi: 10.1177/0031512519846768

Brodsky, W., and Sulkin, I. (2011). Handclapping songs: a spontaneous platform for child development among 5–10-year-old children. *Early Child Dev. Care*. 181, 1111–1136. doi: 10.1080/03004430.2010.517837

Bryant, B. K. (1982). An index of empathy for children and adolescents. *Child. Dev*. 53, 413–425. doi: 10.2307/1128984

Bugos, J., and Jacobs, E. (2012). Composition instruction and cognitive performance: results of a pilot study. *Res. Stud. Music Educ.* 10:2. Available online at: https://commons.lib.jmu.edu/rime/vol10/iss1/2/

Catterall, J. S., and Peppler, K. A. (2007). Learning in the visual arts and the worldviews of young children. *Cambridge J. Educ.* 37, 543–560. doi: 10.1080/03057640701705898

Degé, F., Wehrum, S., Stark, R., and Schwarzer, G. (2011). The influence of two years of school music training in secondary school on visual and auditory memory. *Eur. J. Dev. Psychol.* 8, 608–623. doi: 10.1080/17405629.2011.590668

de Wied, M., Goudena, P. P., and Matthys, W. (2005). Empathy in boys with disruptive behavior disorders. *J Child Psychol Psychiatry*. 46, 867–880. doi: 10.1111/j.1469-7610.2004.00389.x

Elias, M. J., Gara, M., Ubriaco, M., Rothbaum, P. A., Clabby, J. F., and Schuyler, T. (1986). Impact of a preventive social problemsolving intervention on children's coping with middle-school stressors. *Am. J. Community Psychol*. 14, 259–275. doi: 10.1007/BF00911174

François, C., Chobert, J., Besson, M., and Schön, D. (2013). Music training for the development of speech segmentation. *Cereb. Cortex*. 23, 2038–2043. doi: 10.1093/cercor/bhs180

Freeman, G. D., Sullivan, K., and Fulton, C. R. (2003). Effects of creative drama on self-concept, social skills, and problem behavior. *J. Educ. Res.* 96, 131–138. doi: 10.1080/00220670309598801

Guilford, J. P. (1967). Creativity: yesterday, today, and tomorrow. *J. Creat. Behav*. 1, 3–14. doi: 10.1002/j.2162-6057.1967.tb00002

Guo, X., Ohsawa, C., Suzuki, A., and Sekiyama, K. (2018). Improved digit span in children after a 6-week intervention of playing a musical instrument: an exploratory randomized controlled trial. *Front. Psychol.* 8, 1–9. doi: 10.3389/fpsyg.2017.02303

Hightower, A. D., Work, W. C., Cowen, E. L., Lotyczewski, B. S., Spinell, A. P., Guare, J. C., et al. (1986). The Teacher-Child Rating Scale: a brief objective measure of elementary children's school problem behaviors and competencies. *School Psych. Rev.* 15, 393–409. doi: 10.1080/02796015.1986.12085242

Hogenes, M., van Oers, B., Diekstra, R. F. W., and Sklad, M. (2016). The effects of music composition as a classroom activity on engagement in music education and academic and music achievement: a quasi-experimental study. *Int. J.Music. Educ.* 34, 32–48. doi: 10.1177/0255761415584296

Köksal Akyol, A. (2018). Examination of the effect of drama education on multiple intelligence areas of children. *Early Child Dev. Care.* 188, 157–167. doi: 10.1080/03004430.2016.1207635

Kreutzmann, M., Zander, L., and Webster, G. D. (2018). Dancing is belonging! How social networks mediate the effect of a dance intervention on students' sense of belonging to their classroom. *Eur. J. Soc. Psychol*. 48, 240–254. doi: 10.1002/ejsp.2319

Krohne, H. W., Egloff, B., Kohlmann, C. W., and Tausch, A. (1996). Untersuchungen mit einer deutschen Version der "Positive and Negative Affect Schedule" (PANAS) [Investigations with a German version of the Positive and Negative Affect Schedule (PANAS)]. *Diagnostica 42*, 139–156. doi: 10.1037/t49650-000

Lenhard, W., and Lenhard, A. (2016). *Calculation of Effect Sizes.* [Online calculator]. Dettelbach: Psychometrica. Availble online at: https://www.psychometrica.de/effekt_size.html (accessed June 24, 2020).

Mednick, S. (1962). The associative basis of the creative process. *Psychol. Rev.* 69, 220–232. doi: 10.1037/h0048850

Moreno, S., Bialystok, E., Barac, R., Schellenberg, E. G., Cepeda, N. J., and Chau, T. (2011). Short-term music training enhances verbal intelligence and executive function. *Psychol. Sci*. 22, 1425–1433. doi: 10.1177/0956797611416999

Morris, S. B. (2008). Estimating effect sizes from pretest-posttest-control group designs. *Organ. Res. Methods*. 11, 364–386. doi: 10.1177/1094428106291059

Norton, E. S., andWolf,M. (2012). Rapid automatized naming (RAN) and reading fluency: implications for understanding and treatment of reading disabilities. *Annu. Rev. Psychol.* 63, 427–452. doi: 10.1146/annurev-psych-120710-100431

Pekarik, E. G., Prinz, R. J., Liebert, D. E., Weintraub, S., and Neale, J. M. (1976). The pupil evaluation inventory. *J. Abnorm. Child. Psychol*. 4, 83–97. doi: 10.1007/BF00917607

Pelham, W. E., Milich, R., Cummings, E. M., Murphy, D. A., Schaughency, E. A., and Greiner, A. R. (1991). Effects of background anger, provocation, and methylphenidate on emotional arousal and aggressive responding in attention-deficit hyperactivity disordered boys with and without concurrent aggressiveness. *J. Abnorm. Child. Psychol.* 19, 407–426. doi: 10.1007/BF00919086

Rabinowitch, T. C., Cross, I., and Burnard, P. (2013). Long-term musical group interaction has a positive influence on empathy in children. *Psychol. Music*. 41, 484–498. doi: 10.1177/0305735612440609

Rickard, N. S., Bambrick, C. J., and Gill, A. (2012). Absence of widespread psychosocial and cognitive effects of school-based music instruction in 10–13-year-old students. *Int. J. Music. Educ.* 30, 57–78. doi: 10.1177/0255761411431399

Rickard, N. S., Vasquez, J. T., Murphy, F., Gill, A., and Toukhsati, S. R. (2010). Benefits of a classroom based instrumental music program on verbal memory of primary school children: a longitudinal study. *Aust. J. Music Educ*. 1, 36–47.

Roden, I., Grube, D., Bongard, S., and Kreutz, G. (2014a). Does music training enhance working memory performance? Findings from a quasi-experimental longitudinal study. *Psychol. Music.* 42, 284–298. doi: 10.1177/0305735612471239

Roden, I., Könen, T., Bongard, S., Frankenberg, E., Friedrich, E. K., and Kreutz, G. (2014b). Effects of music training on attention, processing speed and cognitive music abilities—findings from a longitudinal study. *Appl. Cogn. Psychol.* 28, 545–557. doi: 10.1002/acp.3034

Roden, I., Kreutz, G., and Bongard, S. (2012). Effects of a school-based instrumental music program on verbal and visual memory in primary school children: a longitudinal study. *Front. Psychol.* 3:572. doi: 10.3389/fpsyg.2012.00572

Roden, I., Zepf, F., d., Kreutz, G., Grube, D., and Bongard, S. (2016). Effects of music and natural science training on aggressive behavior. *Learn. Instr.* 45, 85–92. doi: 10.1016/j.learninstruc.2016.07.002

Schellenberg, E. G., Corrigall, K. A., Dys, S. P., and Malti, T. (2015). Group music training and children’s prosocial skills. *PLoS ONE* 10:e0141449. doi: 10.1371/journal.pone.0141449

Schulz, K. F., and Grimes, D. A. (2002). Sample size slippages in randomized trials: exclusions and the lost and wayward. *Lancet* 359, 781–785. doi: 10.1016/S0140-6736(02)07882-0

Semjen, A., and Vos, P. G. (2002). The impact of metrical structure on performance stability in bimanual 1:3 tapping. *Neurosci. Lett.* 66, 50–59. doi: 10.1007/s004260100073

Sowden, P. T., Clements, L., Redlich, C., and Lewis, C. (2015). Improvisation facilitates divergent thinking and creativity: realizing a benefit of primary school arts education. *Psychol. Aesthet. Creat. Arts.* 9, 128–138. doi: 10.1037/aca0000018

Stangroom, J. (2019). *Social Science Statistics* [Online calculator]. Retrieved from: https://www.socscistatistics.com

Tremblay, R. E., Loeber, R., Gagnon, C., Charlebois, P., Larivée, S., and LeBlanc,M. (1991). Disruptive boys with stable and unstable high fighting behavior patterns during junior elementary school. *J. Abnorm. Child. Psychol*. 19, 285–300. doi: 10.1007/BF00911232

Uanhoro, J. O. (2017). *Effect size calculators* [Online calculator]. Availble online at: https://effect-size-calculator.herokuapp.com/ (accessed June 24, 2020).

Walsh-Bowers, R., and Basso, R. (1999). Improving early adolescents' peer relations through classroom creative drama: an integrated approach. *Child. Sch.* 21, 23–32. doi: 10.1093/cs/21.1.23

Walsh-Bowers, R. T. (1992). A creative drama prevention program for easing early adolescents' adjustment to school transitions. *J. Prim. Prev*. 13, 131–147. doi: 10.1007/BF01325071

Wheeler, V. A., and Ladd, G. W. (1982). Assessment of children's self-efficacy for social interactions with peers. *Dev. Psychol.* 18, 795–805. doi: 10.1037/0012-1649.18.6.795

Zander, L., Kreutzmann, M., West, S. G., Mettke, E., and Hannover, B. (2014). How school-based dancing classes change affective and collaborative networks of adolescents. *Psychol. Sport Exerc*. 15, 418–428. doi: 10.1016/j.psychsport.2014.04.004
